# A Comprehensive Quality Evaluation System for Medicinal Leeches by Integrating Macromolecular Protein Analysis and Small-Molecule Marker Detection as Well as Quantitative Bioassays

**DOI:** 10.3390/ph18060887

**Published:** 2025-06-13

**Authors:** Wenduan Wang, Yufei Liu, Wenjiao Lou, Liangmian Chen, Tianze Xie, Zhimin Wang, Yue Ma, Huimin Gao

**Affiliations:** 1 State Key Laboratory for Quality Ensurance and Sustainable Use of Dao-di Herbs, Institute of Chinese Materia Medica, China Academy of Chinese Medical Sciences, Beijing 100700, China; wangwenduan@126.com (W.W.); lyf820144603202506@163.com (Y.L.); lwj0719@126.com (W.L.); 2 National Engineering Laboratory of Quality Control Technology of Chinese Materia Medica, Institute of Chinese Materia Medica, China Academy of Chinese Medical Sciences, Beijing 100700, China; lmchen@icmm.ac.cn (L.C.); tzxie@icmm.ac.cn (T.X.); zmwang@icmm.ac.cn (Z.W.)

**Keywords:** leech, *Hirudo*, *Whitmania pigra*, *Hirudo nipponica*, antithrombotic bioassay, quality evaluation

## Abstract

**Background/Objectives:** Medical leech (*Hirudo* in the Chinese Pharmacopoeia) is renowned in traditional medicine for its significant antithrombin activity. As an animal-derived medicine with complex and incompletely understood composition, its insufficient quality control measures are met with widespread counterfeiting caused by limited animal resources and rising demand. **Methods:** In this study, an integrated quality evaluation strategy guided by “Totality of the Evidence” (TOE) method is proposed. This strategy combines chemical characterization of small and macromolecular components with bioassays relevant to its clinical functions. A total of 28 batches of samples were analyzed, comprising 23 genuine and 5 counterfeit batches. Species origins were identified by morphology and DNA barcoding. Chemical characterization included TLC, HPLC and UPLC-QTOF-MS/MS for small molecules, and SDS-PAGE with HPLC-Orbitrap Fusion Lumos Tribrid-MS for macromolecules. Antithrombotic activity was assessed by thrombin titration and platelet aggregation assays. **Results:** Several characteristic components were discovered and identified as key quality control markers, including eight small molecules such as an unreported compound SZ-1, plus seven major differential proteins across species. Based on these markers, accurate and rapid authentication methods were established using SDS-PAGE for macromolecules, and both HPLC and TLC for small molecules. Furthermore, using bioassay methods we established for quality evaluation, *Hirudo nipponica* exhibits potent anti-thrombin activity and inhibits platelet aggregation, while *Whitmania pigra* shows weak anti-thrombin activity and promotes platelet aggregation. **Conclusions:** This quality evaluation strategy is not only applicable for the quality assessment of genuine *Hirudo* products of different origins, but also for distinguishing medical leeches from their counterfeits.

## 1. Introduction

Leeches have been used in many cultures for over 2000 years to treat and cure cardiovascular diseases, primarily by promoting blood circulation and preventing blood stasis [1]. Taxonomically, they belong to the kingdom *Animalia*, phylum *Annelida*, and class *Hirudinea*. Approximately 700 leech species have been identified globally, with some being blood-sucking (e.g., *Hirudo medicinalis*) and others non-hematophagous (e.g., *Whitmania pigra* Whitman, WP). In Traditional Chinese medicine (TCM), the dried bodies of medicinal Leech, termed *Hirudo*, were first documented in the Shennong Ben Cao Jing [2]. Although ancient Chinese medical texts predominantly reference blood-sucking leeches, the Chinese Pharmacopoeia (ChP 2020) specifies three official sources: WP, *Hirudo nipponica* Whitman (HN), and *Whitmania acranulata* Whitman (WA). Among these only HN is blood-sucking. Additionally, WA is rarely found in the market and has been mostly substituted by *Whitmania laevis* Whitman (WL) [2]. WP is the most prevalent resource in the TCM market. HN is less common in the TCM market and has largely been substituted by *Poecilobdella manillensis* Lesson (PM) in Guangxi and Yunnan Provinces, as PM complies with local medicinal standards [3]. In recent years, the surging demand for medicinal leeches, coupled with continuous degradation of the environment, has led to the proliferation of more counterfeits, such as *Mimobdella japonica* Blanchard (MJ) and *Poecilobdella javanica* Wahlberg (PJ) [4,5]. This has posed a great challenge to the quality control system of leeches.

In the past, numerous studies convincingly demonstrated that medical leeches exhibit anticoagulant, antithrombotic, anti-atherosclerotic, anti-tumor, anti-inflammatory, anti-fibrotic, and lipid-lowering effects [6]. Nevertheless, the bioactive constituents of medical leeches remain not fully elucidated due to their complex constituents containing small molecules and macromolecules. To date, over 130 compounds have been successfully isolated and identified from medical leeches [6,7,8,9]. These compounds consist of proteins, peptides, pteridines, phosphatidylcholines, glycosphingolipids, and sterols [7]. Among them, hirudin is widely recognized as the principal thrombin inhibitor, which is present in the fresh saliva of leeches [2]. Notably, for application in TCMs, the leeches are scalded, subsequently sun-dried, and finally orally administered as therapeutic. This means that the active ingredients except hirudin need to be further clarified. With the development of omics technology, 1035 proteins and 2323 peptides have been detected in leeches [2,10]. Additionally, 58 proteins and peptides have been isolated, among which 7 proteins and 21 peptides possess anticoagulant activities [6,7,11,12,13]. Several studies have explored potential quality markers for *Hirudo* through proteomics analysis [2,10], but have not been successful in elucidating the anticoagulant active macromolecules and establish the quality control standards. Moreover, for micromolecular research, some pteridines, phosphatidylcholines, and sterols did not exhibit the anticoagulant activity [7]. In view of this, several high-performance liquid chromatography (HPLC) or ultra-performance liquid chromatography (UPLC) methods for quality control have been developed relying on non-characteristic components such as xanthine, hypoxanthine, amino acids, succinic acid and sodium succinate [14,15,16]. Obviously, the existing quality evaluation methods are not fully scientific and rigorous, and there is an urgent and pressing need for efficient quality control methods specifically designed for medicinal leeches.

The Totality of the Evidence (TOE) approach is recommended in the final version of the Botanical Drug Development Guidance for Industry issued by the Food and Drug Administration (FDA) for assessing biosimilar drugs. It pertains to the comprehensiveness of raw material control, chemical testing for quality assessment, and biological assays [17]. Applying TOE to the quality control of animal-derived medicines holds great significance, as it features two prominent highlights. First, it places special emphasis on mass balance (MB) during chemical characterization. Multiple analytical methodologies for various compound classes are emphasized. This is highly suitable for animal-derived medicines, which possess a complex component system comprising both macromolecules and small molecules. Second, bioassays, especially those guided by clinical effects, are emphasized. Bioassays offer the distinct advantage of directly reflecting the therapeutic efficacy of drugs, making them ideal for quantitative evaluation systems to assess the quality of medicines, particularly animal-derived medicines, because it is often challenging to evaluate the quality by means of the quantitative method of chemical markers.

**Figure 1:**
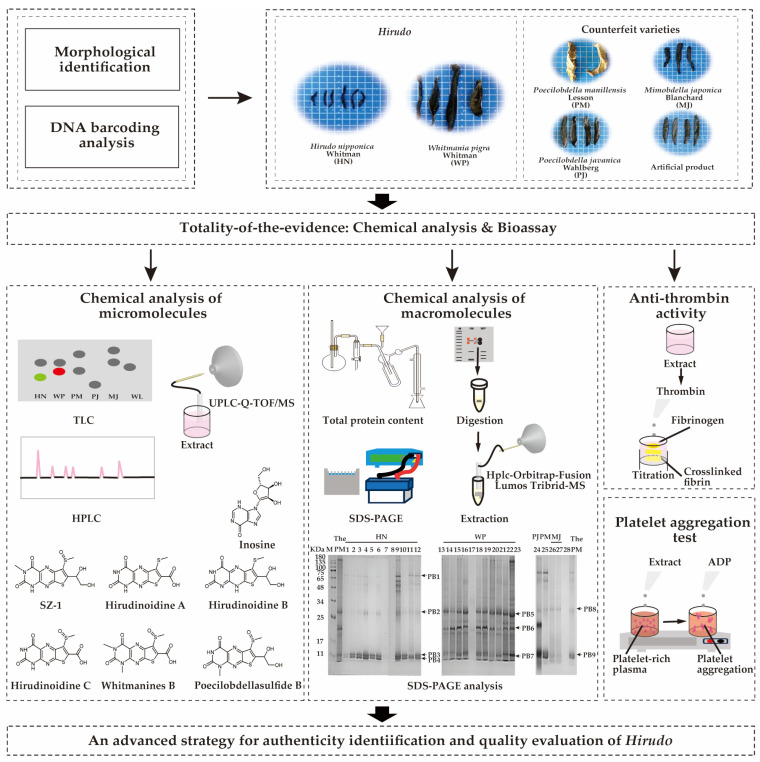
The quality evaluation strategy for medical leeches guided by Totality of the Evidence (TOE).

Therefore, our study under the guidance of TOE, morphological characteristics and DNA barcoding analysis, was employed for species origin identification. HPLC and ultra-performance liquid chromatography quadrupole time-of-flight mass spectrometry (UPLC-QTOF-MS/MS) were employed for chemical characterization of micromolecular aspects. Sodium dodecyl sulfate polyacrylamide gel electrophoresis (SDS-PAGE) and HPLC-Orbitrap Fusion Lumos Tribrid-MS were employed for the chemical characterization of macromolecules. The bioassays, for clinical therapeutic effect, antithrombin and antiplatelet aggregation, were applied to assess the antithrombotic activity. As a result, an efficient quality evaluation strategy for medical leeches integrating comprehensive chemical characterization and antithrombotic bioassays was established. The overall research diagram is shown in Figure 1.

## 2. Results

### 2.1. Identification of Leech Species Based on Morphological Characteristics and DNA Barcoding

**Figure 2:**
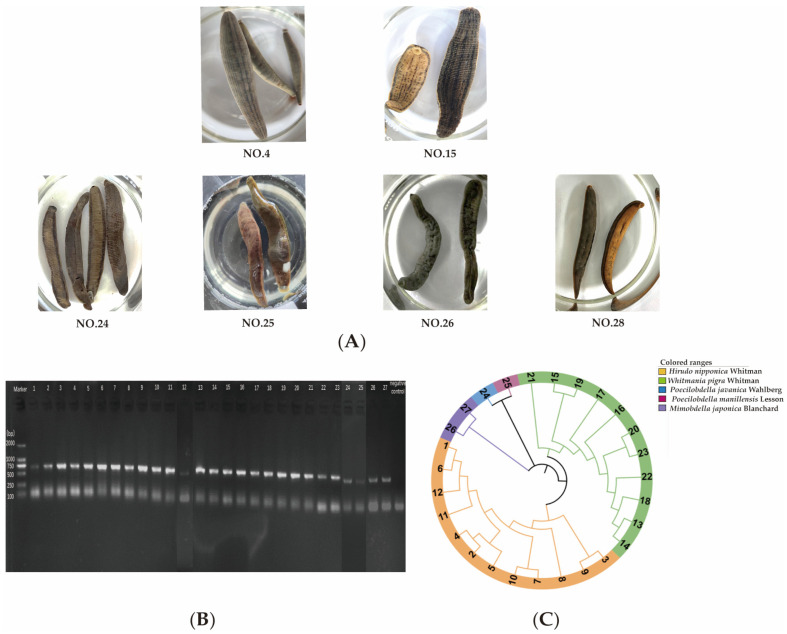
Leech species identification by morphological characteristics and DNA barcoding. (**A**) Characteristic traits of typical samples. (**B**) One percent agarose gel electrophoresis of PCR amplified product of leeches. (**C**) NJ phylogenetic tree of leeches. Samples 1–12: HN, 13–23: WP, 24–28: counterfeits.

In the ChP 2020, the description of dried WP indicates that its body length ranges from 4 to 10 cm and the width is between 0.5 and 2 cm. While dried HN is described that its body length is 2 to 5 cm and the width is 0.2 to 0.3 cm. However, due to variations in species and the mixed use of wild and cultivated products, it has become challenging nowadays to visually differentiate between HN and WP solely based on their body sizes. The primary criterion for identification lies in the characteristic patterns on the dorsal surfaces of different leech species, which only can be observed after soaking the dried whole body. The typical soaking bodies of each leech species were shown in Figure 2A, and the features of different species were described in Table S1. According to these morphological characteristics, 28 batches of samples (detailed information is shown Table S2 and Figure S1) were identified. Among them, samples 1–12 were identified as originating from HN, and samples 13–23 from WP. Samples 24 to 28 were determined to be counterfeits; specifically, sample 24 from PJ, sample 25 from PM, and sample 28 from WL. Samples 26 and 27 remained unidentified. Despite soaking each sample for dozens of hours to reveal the patterns, there are still samples that cannot be identified and require further confirmation.

The cytochrome c oxidase subunit I (COI) gene functions as the core standard DNA barcode for animal species identification, with its utility validated across diverse taxonomic lineages. Here, we applied DNA barcoding technology to analyze COI sequences from 28 batches of leech samples, aiming to achieve precise species authentication. The agarose gel electrophoresis of PCR-amplified products of 28 leech samples is shown in Figure 2B, and the neighbor-joining phylogenetic tree of 28 samples is shown in Figure 2C. The results were largely consistent with those of morphological characteristics, indicating that samples 1 to 12 were from HN, samples 13 to 23 were from WP, and sample 25 was from PM. Additionally, sample 24 was ultimately identified as PJ, and samples 26 and 27 were furtherly identified as MJ. However, the amplification of sample 28 failed. Therefore, counterfeits 24 to 28 were considered as disproof samples.

### 2.2. Micromolecular Chemical Characterization

#### 2.2.1. Chemical Characterization of 28 Samples by HPLC

**Figure 3:**
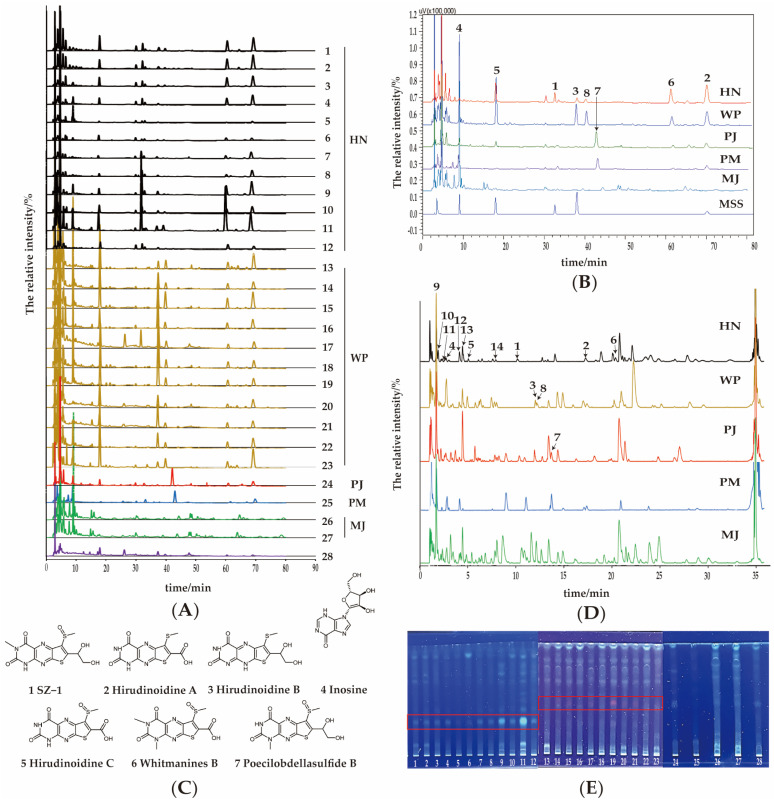
Micromolecular analysis. (**A**) Chromatograms of 23 batches of leech samples and 5 counterfeit samples by HPLC. (**B**) Fitted HPLC characteristic chromatogram of different leech species. (**C**) Structures of the characteristic components. (**D**) BPI chromatograms of representative samples of each leech species by UPLC-QTOF-MS/MS. (**E**) TLC chromatograms of different leech species. MSS: mixed standard solutions, 1–12: HN, 13–23: WP, 24: PJ, 25: PM, 26–27: MJ, 28: unidentified counterfeit.

To develop an HPLC method with reduced requirements for instrumentation and chromatographic columns, we optimized several key aspects of the chromatographic conditions. Specifically, we systematically screened and optimized the mobile-phase composition and elution gradient to achieve optimal separation efficiency. Methanol, acetonitrile, and their combination were evaluated as organic phases. And formic acid, acetic acid, and trifluoroacetic acid (TFA) were added to enhance elution performance. The elution gradient was fine-tuned to balance separation performance and analysis time. These optimizations collectively improved the accuracy and reproducibility of our chromatographic analysis. Ultimately, the optimal conditions entailed a methanol–acetonitrile mixture, TFA in the aqueous phase, and at a relatively low polarity ratio (organic phase increasing from 5% to 20%), yielding the best chromatographic peak elution. Under the optimized conditions, 28 batches of samples were analyzed using HPLC to elucidate the chemical profiles of medical leeches. Their HPLC chromatograms are presented in Figure 3A. Evidently, the chromatographic features of 12 batches of HN samples were largely consistent, with 7 characteristic peaks **1–6** and **8**. Similarly, 10 batches of WP samples were generally uniform, displaying 6 characteristic peaks **2–6** and **8**.

Regarding the counterfeits, samples from PJ and PM exhibited identical sets of 6 characteristic peaks **2–7**. However, a significant difference was observed in the relative abundance of peak **5** between these two species. Peak **5** was notably more prominent in the PJ sample. The chromatograms of MJ samples deviated substantially from those of other species, as they displayed only characteristic peak **4**. Based on the origin of the samples and whether they are cultured or wild, as well as the number of chromatographic peaks and their response intensities, the HPLC profiles of Samples 1 and 15 were selected as characteristic profiles for HN and WP, respectively. Subsequent comparative analysis among HN (Sample 1), WP (Sample 15), and counterfeit samples-PJ (Sample 24), PM (Sample 25), and MJ (Sample 26)-generated representative HPLC profiles, as illustrated in Figure 3B. The chromatographic peaks in the leech samples were identified and assigned by comparing their retention times with those of reference substances, namely hirudinoidine A (peak **2**), hirudinoidine B (peak **3**), hirudinoidine C (peak **5**), and inosine (peak **4**). Their chemical structures are presented in Figure 3C, and the HPLC chromatograms of the mixed reference standards are presented in Figure S1.

#### 2.2.2. Characteristic Compounds Identification in Different Leech Species

To identify more unique components, UPLC-QTOF-MS/MS was employed for analyzing the representative samples of each leech species. The fitted chromatograms of the five leech species intuitively visualized the characteristics, as shown in Figure 3D. Retention time, accurate molecular mass, molecular formula, and the MS/MS fragment ion of compounds was obtained by Masslynx software. Characteristic peaks were qualitatively identified by comparing the retention times with reference standards, analyzing accurate molecular weights, and the possible fragmentation pathways as reported in the literature [18,19]. In total, 13 compounds were identified, including 6 pteridines, 3 nucleosides, 3 amino acids, and 1 piperidine (Table 1).

Nucleosides are a group of nitrogenous substances [20], and three nucleosides (**4**, **9**, and **10**) were identified in our study. The fragmentation pattern of these compounds is characterized by the parent ion of [M + H]^+^, along with typical fragment ions that result from the loss of ribose units (rib), NH_3_, H_2_O, HCN, CO, NH_2_CN, HNCO, and CNCHO [18]. Taking compound **4** as an example, the quasi-molecular ion was represented at *m*/*z* 269.0862 ([M + H]^+^), suggesting a molecular formula of C_10_H_12_N_4_O_5_. The fragment ion at *m*/*z* 137.0445 was generated from loss of a ribose from quasi-molecular ion, followed by a loss of a HCN presented fragment ion at *m*/*z* 110.0673 ([M + H-rib-HCN]^+^). According to the literature [21], compound **4** has been identified as inosine. With the same cracking pattern, compounds **9** and **10** were identified as hypoxanthine and xanthine, respectively.

Pteridines are compounds based on a pyrimido [4,5-b] pyrazine ring [22]. Totally, six pteridines (**1–3**, **5–7**) have been identified based on their mass spectrometric behaviors. According to the reference standards, compounds **2**, **3**, **5** were identified as hirudinoidine A, hirudinoidine B, and hirudinoidine C, respectively. Their fragmentation patterns were elucidated by the parent ion [M + H]^+^, along with typical fragments from the loss of groups such as SCH_3_, SOCH_3_, COOH, CHOHCH_2_OH. Given the similar fragmentation patterns, compound **6**, with a quasi-molecular ion observed at *m*/*z* 355.0169 ([M+H]^+^), was assigned a molecular formula of C_12_H_10_N_4_O_5_S_2_. And the fragment ions at *m*/*z* 293.0145 and *m*/*z* 249.0433 were sequentially formed by the loss of SOCH_3_ and COOH, confirmed the identification of compound **6** as whitmanine B [14,23]. Additionally, compound **7** was assigned as Poecilobdellasulfide B. MS spectra of the fragments and precursor of **1–3**, **5–7** are available in Figures S2–S7.

Moreover, in compound **1**, an unknown component (peak 1), was observed solely in the HN samples with the retention time of 10.18 min and the quasi-molecular ion at *m*/*z* 357.0330 ([M + H]^+^), indicating a molecular formula of C_12_H_12_N_4_O_5_S_2_. Although a similar peak was observed at 31.9 min in WP sample 17, peak **1** was distinct from the interfering peak due to an ultraviolet absorption profile, and identified as a specific compound in HN. Since it was first discovered in this study, no sufficient information was obtained with mass spectrometry to predict its structure. Further isolation has been performed to elucidate its structure.

Compound **1** was obtained as solid and shown to have the molecular formula C_12_H_12_N_4_O_5_S_2_ (nine degrees of unsaturation) by the high-resolution electrospray ionization mass spectrometry (HR-ESI-MS) ion at *m*/*z* 379.0149 [M + Na]^+^ (calcd for C_12_H_12_N_4_O_5_S_2_Na, *m*/*z* 379.0147) (Figure S8). The UV spectrum of **1** (Figure S9) shows a maximum absorption wavelength of 244 nm and 362 nm. The ^1^H NMR (DMSO-*d*_6_, 600 MHz) spectrum of **1** (Figure S10) contains resonances corresponding to two active protons [*δ*_H_ 6.27 (1H, brs), *δ*_H_ 5.11 (1H, brs)], a proton of an oxygenated methylene [*δ*_H_ 5.68 (1H, t, *J* = 5.8 Hz, 13-OH)], a proton of an oxygenated methine [*δ*_H_ 3.65 (1H, d, *J* = 5.7 Hz, 14-OH)], along with two aliphatic methyl signals [*δ*_H_ 3.26 (3H, s, H-10), 3.15 (3H, s, H-12), as shown in Table 2. Analysis of ^13^C NMR (DMSO-*d*_6_, 150 MHz) and HSQC spectra of **1** (Figures S11 and S12) showed twelve carbon signals, including two carbonyl groups [*δ*_C_ 161.9 (C-2), *δ*_C_ 153.7 (C-4)], six unsaturated double bond carbon signals [125.3 (C-4a), 137.5 (C-5a), 128.8 (C-6), 153.2 (C-7), 141.6 (C-8a), *δ*_C_ 157.7 (C-9a)], and an oxygenated methine carbon signal [*δ*_C_ 67.1 (C-13)], an oxygenated methylene carbon signal [*δ*_C_ 66.9 (C-14)]. The remaining two carbon signals were assigned to two methyls [*δ*_C_ 27.8 (C-10), 40.2 (C-12)]. The above-mentioned information was partly similar to that of (*R*)-hirudonucleodisulfide B [24]. Therefore, compound **1** is speculated to be a pteridine derivative. The main differences were the 3-position N atom replaced by methyl group and a 11-position sulfinyl, which were proved by chemical shifts in two methyls [*δ*_H_ 3.26 (3H, s, H-10), 3.15 (3H, s, H-12)].

2D NMR can provide some evidence for the structure of **1** (Figures S12–S14). The ^1^H-^1^H COSY correlations of H-13/H2-14 suggested an adjacent diol structure. The key HMBC correlations (Figure S15) of H-10/C-2/C-4, H-12/C-6, H-13/C-6, H-14/C-7, respectively, verified the substituents at positions N-3, C-6, and C-7. Further, the characteristic absorption peak in IR spectrum (Figure S16) of amino (3239 cm^−1^), methyl (2921 cm^−1^), carbonyl (1728 cm^−1^), carbon nitrogen double bond (1668 cm^−1^), sulfinyl (1594 cm^−1^), double bond (1545 cm^−1^) were similar to that of whitmanine B and (*R*)-hirudonucleodisulfide B [24]. According to this evidence, the structure of **1** was determined as 1-methyl-6-(methylsulfinyl)-7-(1,2-dihydroxyethyl)thieno[3,2-*g*]pteridine-2,4(1*H*,3*H*)-dione, as presented in Figure 3C. It is particularly noteworthy that this compound has fluorescent properties under 365 nm excitation, which makes it extremely useful in species identification by TLC examination.

Furtherly, the fluorescence characteristics of SZ-1 inspired the establishment of a straightforward TLC method centered around characteristic micromolecules for the identification of leech species. A total of 28 samples were analyzed, and their TLC chromatograms are presented in Figure 3E. As a result, a characteristic green fluorescent spot, corresponding to SZ-1, was consistently observed in samples 1–12, but was absent in samples 13–28. Meanwhile, a red fluorescent spot was only present in every WP sample. The finding indicates that the TLC method can effectively distinguish the sources of medicinal leeches. Additionally, the absence of these two fluorescent spots in the counterfeit samples 24–28 further confirmed that by leveraging these green or red spots, this TLC approach can effectively discriminate between HN and WP, and also distinguish genuine medical leeches from counterfeits.

### 2.3. Macromolecular Chemical Characterization

#### 2.3.1. Total Protein Content in Dried Leech Samples and Its Extracts

**Figure 4:**
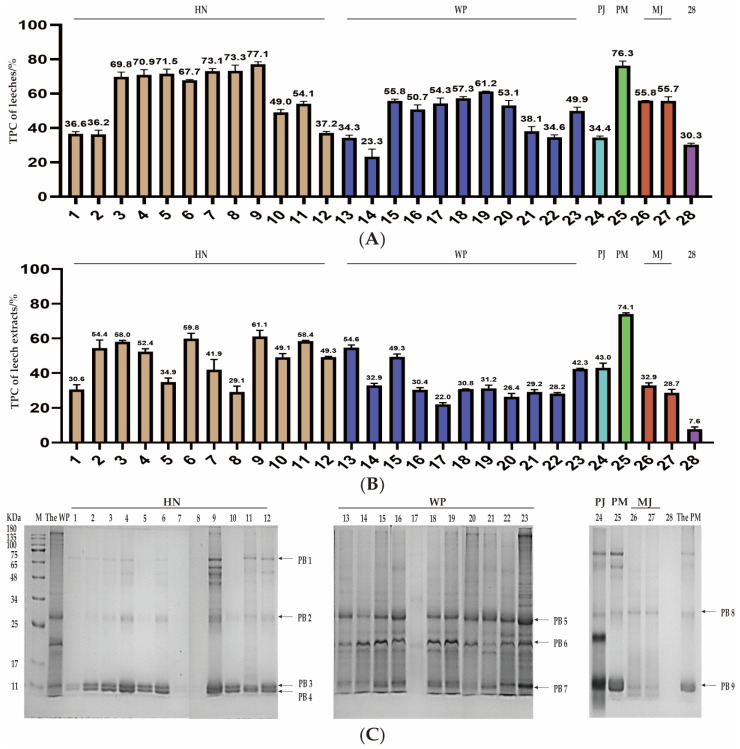
Macromolecules analysis. (**A**) The total protein content of the different leech samples. The numerical value at the top of each column represented the total protein content. (**B**) The total protein content of extracts of the different leech samples. The numerical value at the top of each column represents the total protein content of extracts. (**C**) SDS-PAGE analysis of the 28 leech samples. 1–12: HN, 13–23: WP, 24: PJ, 25: PM, 26–27: MJ, 28: Unidentified counterfeit.

As an important animal-based traditional Chinese medicinal material, the proteins in medical leeches have drawn increasing attention in recent years. To present the protein content of the dried leech powders, each sample was measured by the Kjeldahl method and the results are presented in Figure 4A. The total protein content in HN samples 1–12 ranged from 36.2% to 77.1%. In contrast, the total protein content in WP samples 13–23 was lower, spanning from 23.3% to 61.2%. The total protein content in counterfeit samples showed a polarized outcome. For instance, the protein content in PM sample 25 was as high as 76.3%, while that in PJ sample 24 was as low as 34.4%. The MJ samples 26 and 27 had protein contents of 55.8% and 55.7%, respectively.

Additionally, solubility serves as a fundamental prerequisite for in vivo absorption. Proteins soluble in saline are more likely to blend with digestive fluid in the human gastrointestinal tract, thus establishing favorable conditions for subsequent digestion and absorption. In light of this, the total protein content in saline extract was also measured and the results were shown in Figure 4B. The results clearly demonstrated that the concentration of proteins soluble in extracts was significantly lower than those in the corresponding medicinal materials. Moreover, both dried HN and the extracts of HN exhibited higher the total protein content compared to those of WP. The total protein content in HN extracts ranged from 29.1% to 61.1%, while in WP extracts, it was in a lower range of 22.0% to 54.6%. Regarding the counterfeits, the PM sample 24 had a high value of 74.1%, followed by PJ sample 25 at 43.0%, and MJ samples 26 and 27 had values of 32.9% and 28.7%, respectively. Sample 28 displayed the lowest protein content in both dried body and the extract, being merely 30.3% and 7.6%, respectively. This observation strongly suggests that this counterfeit is more likely to be an artificial product rather than a closely related species of natural leeches.

Additionally, protein content of extracts from various samples provided valuable insights into the influence of processing methods or breeding methods on the potential active proteins in leeches. For both HN and WP, the total protein content of freeze-dried samples is higher than that of sun-dried samples. However, for cultured and wild products, there is not of significant difference in the total protein content of the extracts. It is evident that the growth mode, whether cultured or wild, has no impact on the total protein content, whereas the processing method has a significant impact on the total protein. Obviously, compared with sun-drying, freeze-drying can retain more soluble proteins.

#### 2.3.2. SDS-PAGE Analysis and Distinctive Protein Identification

To characterize the protein distribution, SDS-PAGE analysis was performed on 28 leech samples. The gel electrophoresis profiles of different leech species were presented in Figure 4C. In line with the methodology established in this study, the results disclosed marked divergence in the distribution patterns of major protein bands between HN and WP. In all HN samples, four characteristic protein bands were identified, located at 60–75 kDa (PB1), 25–34 kDa (PB2), and 11 kDa (PB3 and PB4). WP samples exhibited three prominent protein bands, distributed at 25–34 kDa (PB5), 17–25 kDa (PB6), and 11 kDa (PB7). Additionally, the gel electrophoresis of counterfeit samples 24 to 28 demonstrated distinct key protein bands compared with those of HN and WP. This finding indicates that SDS-PAGE can serve as a method to differentiate medical leeches from their counterfeits and to identify the differences between HN and WP. Among the counterfeits, PM samples displayed main protein bands similar to those of HN. PJ presented four major protein bands at 60–75 kDa (PB1), 25–34 kDa (PB2), 17–25 kDa (PB6), and11 kDa (PB7), while MJ showed two principal protein bands at 25–34 kDa (PB8) and 11 kDa (PB9). Notably, sample 28 lacked distinct protein bands, further suggesting that this sample could be pre-extracted.

#### 2.3.3. HPLC-Orbitrap Fusion Lumos Tribrid-MS Analysis of the Distinctive Proteins

Based on the distinctive protein bands discovered by SDS-PAGE analysis, the characteristic proteins were identified by HPLC-Orbitrap Fusion Lumos Tribrid-MS. The protein database downloaded from the UniProt website (https://www.uniprot.org/, accessed on 15 April 2023) was searched, including genus *Hirudo* (453 sequences) and *Whitmania* (82 sequences), family Hirudinidae (612 sequences) and Haemopidae (121 sequences), and class Hirudinea (26,658 sequences). The specific search parameters were set as follows: trypsin enzymatic cleavage, maximum missed cleavage sites set to 2, precursor mass tolerance of 10 ppm, fragment mass tolerance of 0.6 Da, fixed modification of carbamidomethyl (C), and variable modification of oxidation (M).

Protein identification was governed by two key factors, the molecular weight (gel-predicted molecular weights within ±5 kDa) and top-ranked peptide-spectrum match scores. As the results, PB1 was identified as a heat shock protein from *Helobdella robusta*, with a molecular weight (MW) of 70.6 kDa and functions including chaperone activity, apoptosis, autophagy and immunity [25]. PB2 and PB5 were identified as actin, a highly conserved 28.5 kDa protein from *Placobdella parasitica* that is involved in cellular motility and ubiquitously expressed in all eukaryotic cells. PB3 was identified as a HTH psq-type domain-containing protein from *Helobdella robusta* (14.1 kDa), regulating gene expression [26]. PB4 was assigned to neurohemerythrin from *Hirudo medicinalis* (13.8 kDa), which may play a role in the innate immune response of the leech nervous system to bacterial invasion. PB6 aligned with peptidyl–prolyl cis-trans isomerase from *Helobdella robusta* (18.1 kDa), which catalyzes the *cis-trans* isomerization of proline imidic peptide bonds in oligopeptides. PB7 was identified as lysozyme from *Hirudo medicinalis* (14.8 kDa). Lysozymes have catalytic activity and can hydrolyze the (1 → 4)-*β*-linkages between *N*-acetylmuramic acid and *N*-acetyl-D-glucosamine residues in a peptidoglycan and between *N*-acetyl-D-glucosamine residues in chitodextrins. The results demonstrate that PB1-4 and PB5-6 represent first identifications in HN and WP, respectively.

### 2.4. Bioassays of Antithrombotic Activities

#### 2.4.1. Anti-Thrombin Activity Assay

**Figure 5:**
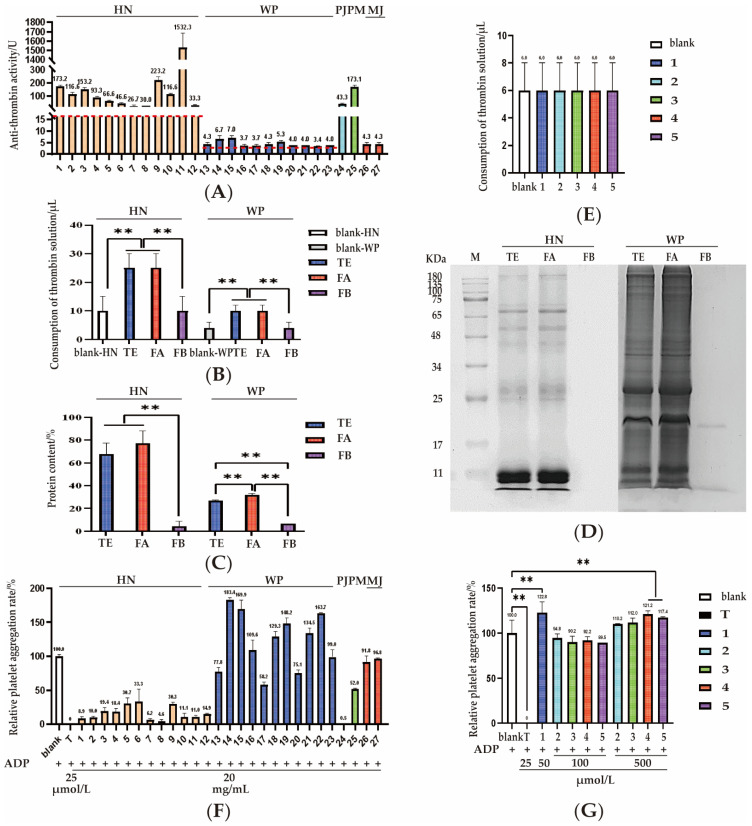
Bioassays of anti-thrombin activity and platelet aggregation. (**A**) The anti-thrombin activity of 27 leech samples. The numerical value at the top of each column represented the anti-thrombin activity. The red line indicates the minimum limits specified in the ChP 2020 for HN and WP. (**B**) The anti-thrombin activity of the various fractions of HN and WP. (**C**) The protein content in various fractions of HN and WP. (**D**) SDS-PAGE analysis of the various fractions of HN and WP. (**E**) Anti-thrombin activity of components 1–5 using WP method. (**F**) The relative platelet aggregation rates of 27 leech samples (20 mg/mL) and ticagrelor (25 μM). The numerical value at the top of each column represented the relative platelet aggregation rate. (**G**) The relative platelet aggregation rate of components **2–5** (100 μmol/L, 500 μmol/L), component **1** (50 μmol/L) and ticagrelor (25 μmol/L). The numerical value at the top of each column represented the relative platelet aggregation rate. The numerical value at the top of each column represented the relative platelet aggregation rate. Relative platelet aggregation rate is defined as the platelet aggregation rate of the samples divided by that of the saline, multiplied by 100%. 1–12: HN, 13–23: WP, 24: PJ, 25: PM, 26–27: MJ. Blank: saline, T: ticagrelor, 1: SZ-1, 2: hirudinoidine A, 3: hirudinoidine B, 4: inosine, 5: hirudinoidine C, **: *p* < 0.01.

Anti-thrombin activity serves as an index for the content determination of *Hirudo* in the ChP 2020 to assess the quality of HN, WP, and WA. Because the anti-thrombin activity of HN is notably higher than that of WP and WA, different concentrations of thrombin solutions are utilized for titration. Specifically, for HN samples, a 40 U/mL thrombin solution was used, whereas WP and WA samples were treated with a 10 U/mL thrombin solution. And different standards are specified: for HN, it should not be lower than 16.0 U, and for WP and WA, it should not be lower than 3.0 U [27]. The results in our study showed that the antithrombin activity of all tested samples, including both medical leeches and the counterfeits, complies with the requirement of content limit above, as respected in Figure 5A. The volume of thrombin consumed by HN samples is significantly higher than that by WP samples, with an average value difference of approximately 50 times. This finding was consistent with the previous report [20] indicating a marked disparity in anticoagulant activity between HN and WP. This significant variability is a crucial factor demanding special consideration in formulating clinical dosages of medications.

Additionally, the 12 batches of HN samples showed a wide range, from 26.7 to 1532.3 U, while the 11 batches of WP samples showed a range of 3.4 to 7.0 U. The discrepancy may be caused by our samples derived from different processing and feeding methods. The freeze-dried products of HN and WP exhibited significantly higher activity compared to their sun-dried products, suggesting that processing methods can influence their anti-thrombin activity; particularly, heating may destroy the components related to the anticoagulant activity in HN and WP. Among the sun-dried samples, wild HN demonstrated markedly greater activity than the cultured HN. In contrast, for WP, no significant difference was detected between wild and cultured products, as both exhibited low anti-thrombin activity. These findings underscore the influence of processing methods and ecological origins on bioactive potency.

On the other hand, it is worth noting that four batches of counterfeit leeches exhibited a wide range of antithrombin activity, from 3.7 to 173.1 U. Specifically, the antithrombin activities of PJ (sample 24), PM (sample 25), and MJ (samples 26 and 27) were 43.3 U, 173.1 U, 4.3 U, and 4.3 U, respectively. Sample 28 was excluded from activity evaluation because it was predicted to be an artificial counterfeit. Remarkably, the anti-thrombin activities of the PM and PJ samples were substantially higher than those of WP, emphasizing a crucial need for stringent quality control to avert the risk of unintended bleeding associated with misuse. Here, what catches our attention is that the volume of thrombin consumed by WP samples is nearly identical to that of the blank. This finding suggests that the anti-thrombin model may lack sufficient accuracy for evaluating WP, and it is essential to investigate an alternative bioassay to achieve a more comprehensive characterization of the anticoagulant activity exhibited by medical leeches.

#### 2.4.2. Correlation Between Composition and Activity

To identify the primary components responsible for anti-thrombin activity, the extracts of representative samples 9 (HN) and 23 (WP) were further fractionated by a 10 kDa ultrafiltration membrane, yielding fractions A (>10 KDa) and B (<10 KDa). For both HN and WP, the total extracts and fraction A consumed equivalent volume of thrombin solution, which was significantly higher than that consumed by fraction B as shown in Figure 5B. This indicates that anti-thrombin activity is predominantly associated with fraction A with molecular weights greater than 10 kDa. For further elucidation of the distribution of active-related proteins, total protein content analysis and SDS-PAGE were performed on the total extracts, fraction A and fraction B. The results were shown in Figure 5C,D. The outcomes indicated that, regarding protein content and the distribution of protein bands, fraction A is basically consistent with the total extract. In conjunction with the anticoagulant activity assessment, it can be concluded that the active molecules are primarily concentrated in Fraction A, the components larger than 10 kDa. And compounds **1–5**, as the characteristic molecules from fraction B, exhibited no anti-thrombin activity (Figure 5E), further indicating that the small molecules in leeches contribute little to anti-thrombin activity, while macromolecules may play a role. This finding holds significant guidance for our subsequent efforts to identify the substances responsible for anticoagulant effect.

#### 2.4.3. Platelet Aggregation

Leech has been widely used in the treatment of cardiovascular diseases due to its antiplatelet aggregation properties by oral administration. Most of these studies were performed in animal models and in clinical practice. Few studies employed platelet aggregation as a bioassay tool. In the present investigation, ADP-induced platelet aggregation is used for assessing antithrombotic efficacy of different leeches 1–27, and the results are shown in Figure 5F. The ADP-induced platelet aggregation rate in the saline blank control was set as 100% and ticagrelor as a positive drug has the relative aggregation rate of 0. The relative platelet aggregation rate of HN samples ranged from 4.6% to 33.3%, whereas WP samples exhibited relative platelet aggregation rates ranging from 75.1% to 183.4%. Compared with the blank group, HN exhibits an inhibition of platelet aggregation, which revealed that the antithrombotic effect of HN has a meaningful impact on both thrombin and platelet aggregation. In contrast, WP samples displayed diverse action trends. Among the tested samples, three samples (13, 17 and 20) demonstrated weak antiplatelet aggregation activity, and the other eight samples exhibited significant promotion of platelet aggregation. These diverse action trends of WP samples posed challenges in high-throughput analysis when employing bioassays as a quality control tool. More efforts have been taken to validate the increased platelet aggregation of WP in vitro. However, notably, this is the first report that the saline extract of WP promotes platelet aggregation, offering crucial insights into the substances conferring leeches’ medicinal properties. Additionally, compared with the blank group, compounds **2–5** showed promotion of platelet aggregation at a concentration of 500 μmol/L. In contrast, compound **1** (SZ-1) exhibited a significant promotion of platelet aggregation at the concentration of 50 μmol/L, as depicted in Figure 5G. This indicates that compound **1** exhibits greater potency in promoting platelet aggregation compared to compounds **2–5**.

When analyzing ADP-induced platelet aggregation across processed products of HN, no significant difference was observed between freeze-dried and sun-dried products. However, for WP, freeze-dried products of WP demonstrated a significantly more pronounced promotion of ADP-induced platelet aggregation compared to the sun-dried. Moreover, in the evaluation of sun-dried products, no statistically significant differences in ADP-induced platelet aggregation were found between wild and cultured specimens of either HN or WP.

The counterfeits showed varying trends in their inhibition of platelet aggregation. Specifically, the relative platelet aggregation rates of the counterfeit samples, including PJ (sample 24), PM (sample 25), and MJ (samples 26 and 27) were 0.5%, 52.0%, 91.8%, and 96.8%, respectively. These results disclosed that PJ, PM, and MJ inhibited platelet aggregation compared with the blank. It was noteworthy that PJ showed a higher level of inhibited platelet aggregation than HN, which aligns well with the activity assessed by the thrombin model. And in both the thrombin and platelet evaluation models, PM showed antithrombotic activity close to the HN samples. These findings indicate that the antithrombotic activity of PJ and PM are comparable to, or even superior to those of HN. This means their abuse poses a risk of bleeding, and there is an even more urgent need for effective quality control methods to ensure medication safety.

## 3. Discussion

Currently, chemical markers are regarded as the mainstream quality control approach for TCMs. However, for animal-derived medicines like *Hirudo*, their effective components remain not fully clarified, posing challenges with relying solely on quantitative evaluation of these components for quality control. In our study, guided by the TOE, an integrated quality evaluation strategy has been established. It includes chemical characterization of both small-molecule and macromolecular components, as well as bioassays that are relevant to the clinical functions. The compounds identified in our study can serve as critical distinguishing markers across species and helped differentiate genuine products from counterfeits, and the quality of the medicinal materials are reflected through their overall anticoagulant activity. The significance of our quality evaluation strategy lies in its comprehensive chemical characterization and bioactive assessment methods for reflecting overall medicinal materials quality levels, rather than solely emphasizing their chemical components.

The TOE approach places special emphasis on mass balance during chemical characterization, particularly for various compound classes. Through systematic analysis of macromolecules and small molecules, we identified characteristic components in leeches, including eight small molecules such as an unreported compound SZ-1, and seven major proteins. This enabled the establishment of rapid and accurate authentication methods for medical leeches via HPLC and TLC, with a focus on small molecular compounds. TLC is a rapid, simple and low-cost identification method to quickly distinguish between HN, WP, and counterfeits. In contrast, besides a clear identification of authentic and counterfeit products, HPLC also determined their species origin and provided more characteristic compound information for each species. This complementary detection system provides rapid and effective identification methods for producers, consumers, and inspectors in real-world applications.

Despite approximately 130 compounds having been reported in leeches, few water-soluble small molecules have been documented. Using our UPLC-QTOF-MS/MS method, 13 compounds were definitively identified in leech samples. Compared with previously reported HPLC-based methods [14,15], our method prioritizes the profiling of hydrophilic components, which are preferentially retained and active in the aqueous decoctions of medical leeches. To resolve higher-polarity molecules, chromatographic conditions were optimized with an organic phase capped at 20%. Notably, in the UPLC-QTOF-MS/MS chromatograms, hirudinoidine A eluted earlier than whitmanine B, while the order was reversed in HPLC analysis due to the difference in acid concentration of the elution system.

The variations in total protein content observed among samples of the same leech species are likely due to differences in their growth stages and body sizes. These kinds of variations in growth stage and body size are quite common in market-collected samples. The SDS-PAGE method in our study allows direct visualization of the differences in the distribution of major macromolecular components among different leech species, thereby providing a rapid and effective approach to discriminate between genuine medicinal leeches and most counterfeits, as well as to differentiate between HN and WP. However, this method was unable to effectively distinguish between HN and PM species. This implies a high degree of protein similarity between HN and PM, likely attributable to their belonging to the same family *Hirudinidae*.

Additionally, Tricine-SDS-PAGE was also performed by us to profile low-molecular-weight proteins (below 10 kDa). The results showed that no clear protein bands below 10 kDa could be observed for any NH and WP samples, including hirudin (7 kDa) [28], which was not detected. As is known, hirudin is the primary component responsible for the anticoagulant effect in fresh blood-sucking leeches and it is exclusively produced in saliva during blood feeding [2,29], which explains why it has never been reported in non-blood-sucking WP [30]. Furthermore, hirudin is rarely detected in *Hirudo*, as evidenced by both our study and previous reports [7]. The absence of hirudin in HN is mainly because the leeches are typically subjected to drying and heat processing during preparation. And hirudin has been proven to be heat-labile [13], meaning its degradation is highly likely during the drying and heat-processing stages.

In our TOE approach, the quality evaluation of *Hirudo* was conducted based on bioassays of the overall medical material. Thrombin and platelets play crucial roles in the intrinsic and extrinsic pathways of blood coagulation. In this study, the two different bioassay methods were assessed to evaluate the quality of multi-batches of leeches, and the results indicated that both methods are effective for quality control. However, each method has its disadvantages and limitations. Thrombin titration is currently recognized as the determination method in the quality standard of *Hirudo* in the ChP 2020 [27]. Our results suggest that it is feasible for the quality evaluation of bloodsucking HN samples, but it is not applicable to non-bloodsucking WP samples, because the consumed thrombin consumption volume of all WP samples showed no significant differences compared to the blank control saline. Although a number of thrombin inhibitors have been reported from WP, their actual in vitro anticoagulation activity is too weak to be used as a quality control index. Instead, the present evidence shows that the platelet aggregation rate can reflect the differences in effect among various batches of HN and WP samples. Therefore, platelet aggregation could be an alternative bioassay method for the quality control of *Hirudo*.

It is worth noting that the results for individual counterfeit samples should be interpreted with caution, due to limitations in the collection of corresponding samples. For qualitative analysis and bioactivity assays, limited samples may lead to less representative characterization results from the variation between samples. However, these restraints pose little challenge to the reliability of this article, as many distinctions revealed by this work are sufficiently significant to be utilized qualitatively. Moreover, we employed multi-dimensional analytical techniques (e.g., HPLC, LC-MS/MS, SDS-PAGE) combined with diverse activity evaluation methods, showing the distinctions of these counterfeit samples as a whole in comparison to the legal species, thereby confirming our findings. Future studies may further validate these results by analyzing multi-batch samples.

## 4. Materials and Methods

### 4.1. Materials, Chemical Reagents

#### 4.1.1. Chemical Reagents

The reference medicinal material of WP and PM were purchased from National Institutes for Food and Drug Control (Beijing, China). Reference substances Hirudinoidine A (purity ≥ 98%) and hirudinoidine B (purity ≥ 98%), were purchased from Chengdu Desite Biological technology Co., Ltd. (Chengdu, China). Hirudinoidine C (purity ≥ 98%) was purchased from Shanghai Nature Standard Technical Service Co., Ltd. (Shanghai, China). Inosine (purity ≥ 99.2%) were purchased from National Institutes for Food and Drug Control (Beijing, China). Biochemical reagents, thrombin (1000 U) and fibrinogen (purity ≥ 93%) were purchased from Solarbio Life Sciences & Technology Co., Ltd. (Beijing, China), adenosine diphosphate (ADP, purity ≥ 98%) was purchased from Beijing Techlink Biomedical Technology Co., Ltd. (Beijing, China), and ticagrelor (purity ≥ 99%) was purchased from MedChemExpress LLC (Monmouth Junction, NJ, USA). Chemical reagents, formic acid (purity > 99%, LC-MS grade), acetonitrile (purity > 99.8%, LC-MS grade), and trypsin (MS grade) were purchased from Thermo Fisher Scientific (Waltham, MA, USA). Unless specified, chemical reagents used in this study were analytical grade.

#### 4.1.2. Materials

A total of 28 batches of dried medical leeches, including wild and cultured, sun-dried and freeze-dried products, were collected from production areas, medicinal materials markets, and commercial suppliers across China.

All the 28 samples were pulverized into powder and sieved through a 50-mesh sieve, and DNA barcode analysis utilized the tissue of the samples. The detailed information for the 28 leech samples is presented in Table S2, and all the leeches’ pictures were shown in Figure S17.

### 4.2. Instruments

HPLC analysis was performed on a Shimadzu LC-20 system (Shimadzu Corporation, Kyoto, Japan) equipped with a hybrid quaternary pump, an autosampler, a diode array detector, and an online degasser. UPLC-QTOF-MS/MS analysis was conducted using a Waters ACQUITY UPLC H-Class system (Waters, Milford, MA, USA) connected to an Xevo G2-S Q-TOF mass spectrometer via an electrospray ionization (ESI) interface. TLC analysis was carried out with an Automatic TLC Sampler 4 (Camag, Muttenz, Switzerland). Protein profiles were analyzed by HPLC-Orbitrap Fusion Lumos Tribrid-MS (Thermo Fisher Scientific, Waltham, MA, USA). Platelet aggregation was determined using an LBY-NJ4 Platelet Aggregometer (Beijing Techlink Biomedical Technology Co., Ltd., Beijing, China). IR spectra were measured on a VERTEX 70V Fourier transform infrared (FTIR) spectrometer (Bruker, Faellanden, Switzerland). NMR spectra were recorded on an Aglient DD2 600 MHz spectrometer (Aglient, Santa Clara, CA, USA).

### 4.3. Samples Preparation

Each powdered sample (0.5 g) was extracted with 10 mL of 50% methanol for 30 min under sonication, and then the loss weight was made up with solvent. The extracted solution was filtered and centrifuged (12,000 rpm, 10 min) for HPLC and UPLC-Q-TOF/MS analysis. The mixed standard solutions of four reference compounds were prepared in 50% methanol at the following concentrations (μg/mL): hirudinoidine A (31.18), hirudinoidine B (30.59), hirudinoidine C (30.00), and inosine (33.59).

Each sample powder (1.0 g) was accurately weighed and extracted with 5 mL of 95% ethanol under sonication for 30 min. The extracted solution was filtered and centrifuged (12,000 rpm, 10 min) for TLC analysis.

Each powdered sample (1.0 g) was extracted with 5 mL of saline in a shaker for 30 min at room temperature. The extracted solutions were centrifuged at 12,000 rpm for 10 min. The supernatants were used for total protein determination, SDS-PAGE analysis and anti-thrombin bioassays [27]. Solutions of the reference medicinal material of WP and PM were prepared following the same method as the sample solutions. Stock solution of the four reference compounds hirudinoidine A, hirudinoidine B, hirudinoidine C, and inosine were solved in dimethyl sulfoxide (DMSO) at a concentration of 25 mmol/L, separately. And each stock solution was diluted with saline to a concentration of 100 μmol/L for the determination of anti-thrombin activity.

Each saline extract solution was lyophilized, and reconstituted with water at 200 mg/mL for platelet aggregation assays. The positive control drug, ticagrelor was solved in DMSO at a concentration of 6.25 mmol/L, and diluted in platelet-rich plasma (PRP) to a concentration of 25 μmol/L. Additionally, Stock solution of the compounds hirudinoidine A, hirudinoidine B, hirudinoidine C, and inosine was diluted with PRP to a concentration of 100 μmol/L and 500 μmol/L for the determination of platelet aggregation assay.

### 4.4. Morphological Characteristics and DNA Barcoding Analysis

#### 4.4.1. Morphological Characteristics

To observe morphological characteristics, samples were soaked individually in water until the coloration and texture of the dorsal and ventral surfaces became evident. Post-soaking, samples were examined under a magnifying glass to document features.

#### 4.4.2. DNA Barcoding Analysis

DNA barcoding analysis was performed according to literature [31]. For total DNA extraction, 35 mg of tissue from each sample was used after being ground into powder. Total DNA of each sample was extracted according to the method of the DNA extraction kit (TIANamp Genomic DNA Kit DP304 (TIANGEN, Beijing, China)). PCR amplification was performed in 20 μL reaction mixture containing 10 μL (2×) PCR Master Mix, 1.0 μL of each primer (with the specific primers based on the COI sequence), 1 μL genomic DNA, and 8 μL ddH_2_O. For DNA amplification, an initial denaturation at 94 °C for 1 min was performed followed by 5 cycles at 94 °C for 1 min, 45 °C annealing for 1.5 min; then extension at 72 °C for 1.5 min; and 35 cycles at 94 °C for 1 min, 50 °C annealing for 1.5 min; then extension at 72 °C for 1 min; and a final elongation step at 72 °C for 5 min. After detection by 1% agarose gel electrophoresis subjected to the PCR product, Sanger sequencing was conducted by SinoGenoMax. The specific primers based on the COI sequence (Sequencing forward primer: 5′-GGTCAACAAATCATAAAGATATTGG-3′, Sequencing reverse primer: 5′-TAAACTTCAGGGTGACCAAAAAATCA-3′) were synthesized by Beijing Liuhe BGI Co., Ltd. (Beijing, China).

### 4.5. Micromolecules Analysis by HPLC and UPLC-QTOF-MS/MS

#### 4.5.1. HPLC Analysis Condition

The HPLC analyses were performed on a Shimadzu LC-20 system equipped with a hybrid quaternary pump, an autosampler, a diode array detector, and an on-line degasser. A Kromasil C_18_ column (250 mm × 4.6 mm, 5 μm, AkzoNobel, Bohus, Sweden) was used for the separation. The linear gradient elution was performed with 0.05% trifluoroacetic acid-water solution as mobile phase A and methanol-acetonitrile (4:1, *v*/*v*) solution as mobile phase B. The elution program was 0–5 min, 5–9% B; 5–20 min, 9–9% B; 20–22 min, 9–14% B; 22–40 min, 14–14% B; 40–42 min, 14–20% B; 42–80 min, 20–20% B. The detection wavelengths were 245 nm, the column temperature was 30 °C, the flow rate was 1.0 mL/min, and the injection volume was 10 μL.

#### 4.5.2. UPLC-QTOF-MS/MS Analytical Conditions

The UPLC-QTOF-MS/MS analysis was performed on a Waters ACQUITY UPLC H-CLASS system connected to an Xevo G2-S Q-TOF mass spectrometer via an electrospray ionization (ESI) interface. A Waters BEH C_18_ column (100 × 2.1 mm, 1.7 μm, Waters, Milford, MA, USA) with a column temperature maintained at 30 °C was used for the separation. A binary gradient elution system was composed of 0.05% trifluoroacetic acid–water solution (A) and methanol–acetonitrile (4:1, *v*/*v*) solution (B) with gradient elution system as follows: 0–2 min, 5–9% B; 2–8 min, 9–9% B; 8–9 min, 9–14% B; 9–16 min, 14–14% B; 16–17 min, 14–20% B; 17–32 min, and 20–20% B. The flow rate was 0.2 mL/min.

All MS data were acquired in positive ion mode. The ion-source parameters were set as follows: source temperature, 120 °C; desolvation temperature, 450 °C; desolvation gas (N_2_), 800 L/h; capillary voltage, 2.2 kV; cone voltage, 40 V; and MS scan range, *m*/*z* 100–1200. And all MS data was acquired using the MassLynx 4.1 software.

### 4.6. Isolation of SZ-1

The raw material of the HN (Sample 11, 150.0 g) was smashed and extracted in 95% ethanol by ultrasound for 30 min (5 × 1.5 L). The extracting solution was centrifuged with a centrifuge and the supernatant was concentrated. The extract (5.7 g) was separated by MCI column chromatography with methanol–water (20:80~0:100) and ten fractions were obtained; Fr.1~Fr.10. Fr.1 (1.4 g) was then separated by Sephadex LH-20 column with methanol–water (1:1, *v*/*v*) to obtain Fr.1-1~Fr.1-4. Fr.1-2 (462.3 mg), which was fractionated by silica gel column and eluted successively with a CH_2_Cl/MeOH (100:0~0:100) to divide into Fr.1-2-1~Fr.1-2-7, and Fr.1-2-4 (76.4 mg) was processed by semi-preparative HPLC (244 nm) to yield SZ-1 (*t*_R_ = 17.2 min; 3.60 mg; CH_3_CN/H_2_O 23:77). Using HPLC to give a pure SZ-1 with purify higher than 98% (Figure S18). IR spectra were measured on a VERTEX 70V Fourier transform infrared (FTIR) spectrometer (Bruker, Faellanden, Switzerland). NMR spectra were recorded on an Aglient DD2 600 MHz spectrometer (Aglient, Santa Clara, CA, USA).

### 4.7. TLC for Identification

TLC was performed on 10 × 20 cm TLC Silica gel G glass-backed plates (20230221) from Qingdao Marine Chemical (Qingdao, Shandong, China). Samples of leech extracts were spotted onto TLC plates using an Automatic TLC Sampler 4. TLC plates were developed with chloroform–methanol (8:1, *v*/*v*) and observed under UV light at 365 nm.

### 4.8. Total Protein Content in Dried Leech and Total Extracts

The dried leech powder (5 mg) of each sample was accurately weighed and analyzed by the Kjeldahl method to gain the protein content in dried leech [32]. The dried leech powder (1.0 g) was extracted with saline in a shaker for 30 min at room temperature, with centrifuged (12,000 rpm, 10 min) and freeze-dried. The dried extract (5 mg) of each sample was accurately weighed and analyzed by the Kjeldahl method to obtain the protein content in total extracts.

To complete the process, the following steps can be taken: Accurately weigh 5 mg of each sample into a Kjeldahl flask. Add 0.3 g of K_2_SO_4_, 5 drops of 30% CuSO_4_ solution, and 2.0 mL of H_2_SO_4_. Heat the mixture gently until effervescence diminishes, then increase the temperature until a clear green solution is obtained. Maintain boiling for 10 min, cool, and add 2.0 mL of water. Transfer the digested mixture and 10 mL of 40% NaOH solution to a Kjeldahl distillation apparatus. Steam-distill the liberated ammonia into 10 mL of 2% H_3_BO_3_ solution containing 5 drops of methyl red–bromocresol green mixed indicator. Titrate the distillate with 0.005 mol/L H_2_SO_4_ until the endpoint (color transition from blue-green to grayish purple). Perform a blank test (omitting the sample) under identical conditions for correction. Record the volume of the sulfuric acid titrant (0.005 mol/L) consumed and calculate according to the following formula:

N = (V_1_ − V_2_) × 0.1041 × 6.25/m × 100% (1)

In the formula: N represents the protein content units per 1 mg, with the unit of %; V_1_ is the volume of the sulfuric acid titrant (0.005 mol/L) consumed by the sample, in mL; V_2_ is the volume of the sulfuric acid titrant (0.005 mol/L) consumed by the blank test, in mL; m is the sample weight, with the unit of mg.

### 4.9. Protein Profiles by SDS-PAGE and LC-MS/MS

#### 4.9.1. SDS-PAGE Analysis

Saline supernatants of leech samples were diluted by a factor of 10 to 100 to obtain a detection solution with an appropriate concentration for SDS-PAGE analysis. The SDS-PAGE comprised a 12% (*w*/*v*) separating gel and a 5% (*w*/*v*) stacking gel. A rainbow 180 broad range protein marker from Solarbio Life Sciences (ranging 11–180 kDa) was used and the gels were stained with Coomassie Brilliant Blue.

#### 4.9.2. LC-MS/MS Acquisition

Main differential protein bands from HN and WP were excised from the gels, and transferred to microtubes. The in-gel digestion followed an advanced protocol [33]. Protein-containing gel lanes were cut into 1 × 1 mm cubes, incubated with 50% acetonitrile and 50 mM ammonium bicarbonate at 37 °C for 1 h, and underwent reduction and alkylation using 10 mM DTT and 55 mM IAA. The cubes were washed, dehydrated, and air-dried, then rehydrated and digested overnight at 37 °C in 50 mM ammonium bicarbonate with 0.5 μg/mL trypsin. Digested peptides were extracted, desalinated on a C_18_ column, dehydrated by Speedvac, dissolved in 0.1% formic acid/1% acetonitrile aqueous solution, and analyzed by HPLC-Orbitrap Fusion Lumos Tribrid-MS. MS raw files were processed and analyzed with Proteome Discoverer 2.4 (Thermo Fisher Scientific) and Mascot search engine (version 2.6.1).

### 4.10. Anti-Thrombin Activity

The anti-thrombin activity of leech samples was measured following the method for *Hirudo* outlined in the ChP (2020 edition) [27]. Saline supernatants (100 μL) were transferred to a test tube (8 × 38 mm), added 200 μL of freshly prepared Tris-HCl buffer (pH 7.4) containing 0.5% (*w*/*v*) bovine fibrinogen. The mixture was incubated at 37.0 ± 0.5 °C for 5 min. For HN, thrombin solution (40 U/mL) was titrated dropwise (5 μL per addition at 1 min intervals, gently shaking the tube while adding drops) until clot formation; For WP, thrombin solution (10 U/mL) was similarly titrated (2 μL per addition at 4 min intervals). The total thrombin volume required for coagulation was recorded, and anticoagulant activity was calculated using the formula:

U = C_1_V_1_/C_2_V_2_ (2)

In the formula: U represents the anti-thrombin activity units per 1 g, with the unit as U/g; C_1_ is the concentration of the thrombin solution, in μ/mL; C_2_ is the concentration of the test sample solution, in g/mL; V_1_ is the volume of the thrombin solution consumed, in μL; V_2_ is the volume of the test sample solution added, in μL.

### 4.11. Adenosine Diphosphate (ADP)-Induced Platelet Aggregation Test

Blood was collected from the abdominal aorta of anesthetized rats, centrifuged at 1200 rpm for 10 min at room temperature. The resulting platelet-rich plasma (PRP) supernatant was removed. The residue was centrifuged at 4000 rpm for 10 min at room temperature to obtain platelet-poor plasma (PPP). The PRP was adjusted with PPP in order to obtain platelet counts of 400 × 10^9^ L.

Platelet aggregation was determined by Born’s turbidimetric method using LBY-NJ4 Platelet-Aggregometer. Briefly, PRP (270 μL) was pre-incubated with vehicle, positive control or different samples (30 μL) for 4 min at 37 °C, followed by the addition of 6 μL of Adenosine diphosphate to induce the platelet aggregation. The maximum aggregation rate (MAR) was recorded within 5 min at 37 °C.

### 4.12. Statistical Analysis

For statistical analysis, the data acquired from independent experiments were presented as mean ± SD. SAS software (version 9.4) was used for analysis. ANOVA analysis of variance was used for comparison of multiple groups of data, and unpaired *t* tests were used for comparison of the two groups of data. Differences were considered significant at *p* < 0.01.

## 5. Conclusions

In this study, we developed an advanced quality evaluation strategy for medical leeches under the guidance of the TOE. On the basis of accurate species identification by morphological characteristics and DNA barcoding, a comprehensive chemical analysis of multi-batch leech samples, covering both micromolecules and macromolecules, was conducted. Several characteristic compounds were identified for the first time−specifically, seven proteins and an unpublished pteridine derivative. These components were further confirmed to be markers for distinguishing various leech species. Notably, efficient methods using SDS-PAGE, HPLC or TLC were successfully developed for rapid authenticity authentication. Additionally, antithrombin activity and platelet aggregation were employed as two bioassay methods to evaluate the quality of medicinal leeches. This quality evaluation strategy, combining chemical characterization with bioassays, represents the attempt to apply the TOE approach in the quality control of TCM, and our outcomes demonstrated its substantial enlightening significance and practical value.

## Figures and Tables

**Table 1:** Thirteen Compounds identified by UPLC-QTOF-MS/MS.

Name	RT (min)	[M + H]^+^ Detected	[M + H]^+^ Expected	Error (ppm)	Formula	Fragments	Leech Species				
							HN	WP	PJ	PM	MJ
hypoxanthine (**9**)	1.68	137.0459	137.0463	−2.9	C_5_H_4_N_4_O	119.0358, 110.0345	+	+	+	+	+
xanthine (**10**)	1.86	153.0408	153.0413	−3.3	C_5_H_4_N_4_O_2_	110.0340	+	+	+	+	+
leucine (**11**)	2.47	132.1019	132.1025	−4.5	C_6_H_13_NO_2_	113.9624, 86.0943	+	+	+	+	+
inosine (**4**)	2.73	269.0862	269.0886	−8.9	C_10_H_12_N_4_O_5_	137.0445, 110.0673	+	+	+	+	+
2-piperidone (**12**)	4.10	100.0755	100.0762	−7.0	C_5_H_9_NO	82.0647, 72.0768,	+	+	+	+	+
phenylalanine (**13**)	4.41	166.0862	166.0868	−3.6	C_9_H_11_NO_2_	149.0216, 120.0800, 103.0536, 91.0529	+	+	+	+	+
hirudinoidine C (**5**)	5.02	326.9858	326.9858	0	C_10_H_6_N_4_O_5_S_2_	264.9786, 219.9649	+	+	+	+	−
tryptophan (**14**)	7.89	205.0962	205.0977	−7.3	C_11_H_12_N_2_O_2_	188.0751, 146.0580, 118.0643, 91.0531	+	+	+	+	+
unknown (**1**)	10.18	357.0330	357.0327	0.8	C_12_H_12_N_4_O_5_S_2_	295.0060, 234.9884	+	−	−	−	−
hirudinoidine B (**3**)	11.97	327.0209	327.0222	−4.0	C_11_H_10_N_4_O_4_S_2_	281.0528, 221.9807	+	+	+	+	−
poecilobdellasulfide B (**7**)	13.71	357.0320	357.0327	−2.0	C_12_H_12_N_4_O_5_S_2_	294.0190, 235.0243	−	−	+	+	−
hirudinoidine A (**2**)	17.32	310.9910	310.9909	0.3	C_10_H_6_N_4_O_4_S_2_	264.9838, 219.9634	+	+	+	+	−
whitmanine B (**6**)	20.44	355.0169	355.0171	−0.6	C_12_H_10_N_4_O_5_S_2_	293.0145, 249.0433	+	+	+	+	−

**Table 2:** ^1^H (*J* in Hz, 600 MHz) and ^13^C NMR data (150 MHz) of SZ-1 in DMSO-*d*_6_.

Position	*δ*_H_	*δ*_C_	Position	*δ*_H_	*δ*_C_
2	-	161.9	9a	-	157.7
4	-	153.7	13	5.68, t (*5.8*)	67.1
4a	-	125.3	14	3.65, d (*5.7*)	66.9
5a	-	137.5	N_3_-CH_3_	3.26	27.8
6	-	128.8	-CH_3_	3.15	40.2
7	-	153.2	13-OH	6.27, brs	-
8a	-	141.6	14-OH	5.11, brs	-

## Data Availability

The data presented in this study are available within the article, the associated Supplementary Materials, or on request from the authors.

## Supplementary Materials

The following supporting information can be downloaded at https://www.mdpi.com/article/10.3390/ph18060887/s1: Figure S1: The HPLC profile of a mixture of reference substance SZ-1, hirudamine A, hirudamine B, hirudamine C, and inosine; Figures S2–S7: MS spectrum of the fragments and precursor of 1–3, 5–7; Figure S8: *HR-ESI-MS* spectrum of SZ-1; Figure S9: UV spectrum of SZ-1; Figure S10: *^1^H NMR spectrum* of SZ-1 in DMSO-d_6_; Figure S11: *^13^C NMR spectrum* of SZ-1 in DMSO-d_6_; Figure S12: *HSQC spectrum* of SZ-1 in DMSO-d_6_; Figure S13: *^1^H-^1^H COSY spectrum* of SZ-1 in DMSO-d_6_; Figure S14: *HMBC spectrum* of SZ-1 in DMSO-d_6_; Figure S15: Key *^1^H-^1^H COSY* and *HMBC* correlations of SZ-1; Figure S16: *IR spectrum* of SZ-1; Figure S17: The leeches’ pictures; Figure S18: HPLC spectrum of SZ-1; Table S1: The specific characteristic features of each leech species; Table S2: The detailed information of the 28 leech samples.

## References

[1] Wu S.H., Zhou Y.Y., Wang Y., Zhang Z.P. (2024). Therapeutic Potentials of Medicinal Leech in Chinese Medicine. Am. J. Chin. Med..

[2] Li F.G., Shi X.Y., Yang L., Lu X., Qi Y., Li P., Yang H., Gao W. (2024). Quantitative proteomics based bioactive proteins discovery and quality control of medicinal leeches. J. Ethnopharmacol..

[3] Meng F.M., Liu Z.C., Sun J.W., Kong D.J., Wang Y.X., Tong X.R., Cao Y.R., Bi X.X. (2022). Insights into gut microbiota communities of *Poecilobdella manillensis*, a prevalent Asian medicinal leech. J. Appl. Microbiol..

[4] Shi C.F., Guo Y.H., Yao L.J., Xu Y.H., Zhou J., Hua M.L. (2025). Development of a mitochondrial mini-barcode and its application in metabarcoding for identification of leech in traditional Chinese medicine. Sci. Rep..

[5] Ma S.C., Wei F. (2019). Handbook of Traditional Identification of Chinese Medicinal Materials.

[6] Jiang Q., Wang L.N., Liu Q., Yang C.M., Zhang Y.Q. (2022). Research progress on processing history evolution, chemical constituents and pharmacological action of *Hirudo*. China J. Chin. Mater. Med..

[7] Dong H., Ren J.X., Wang J.J., Ding L.S., Zhao J.J., Liu S.Y., Gao H.M. (2016). Chinese Medicinal Leech: Ethnopharmacology, Phytochemistry, and Pharmacological Activities. Evid.-Based Complement. Altern. Med..

[8] Huang Q.Y., Gao Q., Chai X.X., Ren W., Zhang G.F., Kong Y.J., Zhang Y., Gao J.P., Lei X.X., Ma L. (2020). A novel thrombin inhibitory peptide discovered from leech using affinity chromatography combined with ultra-high performance liquid chromatography-high resolution mass spectroscopy. J. Chromatogr. B.

[9] Zheng Y.Z., Ji X.R., Liu Y.Y., Jiang S., Zhang J.Q., Zhang J.L., Kong Y. (2021). WPK5, a novel kunitz-type peptide from the leech *Whitmania pigra* inhibiting factor XIa, and its loop-replaced mutant to improve potency. Biomedicines.

[10] Liao J.M., Gao M., Ding Y.L., Bi Q.R., Huang D.D., Luo X.X., Yang P.L., Li Y., Huang Y., Yao C.L. (2023). Characterization of the natural peptidome of four leeches by integrated proteogenomics and pseudotargeted peptidomics. Anal. Bioanal. Chem..

[11] Ren S.H., Liu Z.J., Cao Y., Hua Y., Chen C., Guo W., Kong Y. (2019). A novel protease-activated receptor 1 inhibitor from the leech *Whitmania pigra*. Chin. J. Nat. Med..

[12] Hu B., Xu L.X., Li Y., Bai X., Xing M.C., Cao Q., Liang H., Song S.L., Ji A.G. (2020). A peptide inhibitor of macrophage migration in atherosclerosis purified from the leech *Whitmania pigra*. J. Ethnopharmacol..

[13] Zhang Y.J., Yang R., Wang L.W., Li Y., Han J., Yang Y.Y., Zheng H.X., Lu M.Y., Shen Y.P., Yang H. (2022). Purification and characterization of a novel thermostable anticoagulant protein from medicinal leech *Whitmania pigra* Whitman. J. Ethnopharmacol..

[14] Wu R.S., Xie F.F., Wu M.L., Qiu Y.J., Li G.W., Tong P.Z., Luo W.H. (2024). Quality Evaluation of Leeches from Different Sources Based on Characteristic Mapping and Biological Activity. J. Chin. Mater. Med..

[15] Li G.W., Qiu Y.J., Tong P.Z., Hu Q.P., Deng L.P., Yang L., Sun D.M. (2023). Quality analysis of *Hirudo* with different origins and its adulterants. Nat. Prod. Res. Dev..

[16] Liu W.Z., Fan J.W., Li Y.F., Qiu X.J., Liu X.D., Su R.Q., Zhao Z.Q. (2014). Simultaneous determination of seven components in dried *Whitmania pigra* by HPLC. Chin. J. Pharm. Anal..

[17] U.S. Food and Drug Administration https://www.fda.gov/regulatory-information/search-fda-guidance-documents/botanical-drug-development-guidance-industry.

[18] Zhang F.Y., Li B.C., Wen Y., Liu Y.Y., Liu R., Liu J., Liu S., Jiang Y.P. (2022). An integrated strategy for the comprehensive pro-filing of the chemical constituents of *Aspongopus chinensis* using UPLC-QTOF-MS combined with molecular networking. Pharm. Biol..

[19] Liang C.R., Yao Y.Q., Ding H.R., Li X.M., Li Y.B., Cai T. (2022). Rapid classification and identification of chemical components of Astragali radix by UPLC-Q-TOF-MS. Phytochem. Anal..

[20] Zhang F.C., Fei Q.Q., Huang X.J., Yu S., Qiu R.L., Guan L., Wu B.X., Shan M.Q. (2024). LC-MS based strategy for chemical profiling and quantification of dispensing granules of *Ginkgo biloba* seeds. Heliyon.

[21] Feng Y.J., You X.J., Ding J.H., Zhang Y.F., Yuan B.F., Feng Y.Q. (2022). Identification of Inosine and 2′-O-Methylinosine Modifications in Yeast Messenger RNA by Liquid Chromatography-Tandem Mass Spectrometry Analysis. Anal. Chem..

[22] Martinez V.C., Alcaraz A.J.R., Vera M., Guirado A., Esparza M.M., Penarrubia P.G. (2019). Therapeutic potential of pteridine derivatives: A comprehensive review. Med. Res. Rev..

[23] Li T., Wang G.C., Wang C.H., Ye W.C. (2013). Three New Pteridines from the Leech *Whitmania pigra*. Chem. Lett..

[24] Li T. (2013). Chemical Constituents from *Whitmania pigra*. Master’s Thesis.

[25] Milani A., Basirnejad M., Bolhassani A. (2019). Heat-shock proteins in diagnosis and treatment: An overview of different biochemical and immunological functions. Immuntherapy.

[26] Li C.X., Liao L.S., Wan X.D., Zhang F.F., Zhang T., Luo X.M., Zhao S., Feng J.X. (2021). PoxCbh, a novel CENPB-type HTH domain protein, regulates cellulase and xylanase gene expression in *Penicillium oxalicum*. Mol. Microbiol..

[27] Chinese Pharmacopoeia Committee (2020). Pharmacopoeia of People’s Republic of China Volume 1.

[28] Huang R.H., Shu Q., Liang S.P. (1998). Purification and Identification of Native Hirudin From the Digestive Juice of Leeches. Acta Sci. Nat. Univ. Norm. Hunan.

[29] Zhang W., Zhang R.X., Li J., Liang F., Qian Z.Z. (2013). Species study on Chinese medicine leech and discussion on its resource sustainable utilization. Chin. J. Chin. Mater. Med..

[30] Kui H.Q., Lei Y., Jia C.X., Xin Q.C., Tursun R., Zhong M., Liu C.X., Yuan R.J. (2024). Antithrombotic pharmacodynamics and metabolomics study in raw and processed products of *Whitmania pigra* Whitman. Heliyon.

[31] Chen S.L. (2015). Standard DNA Barcodes of Chinese Matera Medica in Chinese Pharmacopoeia.

[32] Chinese Pharmacopoeia Committee (2020). Pharmacopoeia of People’s Republic of China Volume 4.

[33] Li X.X., Chu Z., Feng C.R., Song P., Yang T., Zhou L.R., Zhao X., Chai X., Xing J.L., Chen S. (2024). Unveiling the molecular mechanisms of size-dependent effect of polystyrene micro/nano-plastics on Chlamydomonas reinhardtii through proteomic profiling. Chemosphere.

