# Supplementary material for: A Comprehensive Quality Evaluation System for Medicinal Leeches by Integrating Macromolecular Protein Analysis and Small-Molecule Marker Detection as Well as Quantitative Bioassays
Source: Pharmaceuticals (Basel). 2025 Jun 13;18(6):887. doi: 10.3390/ph18060887 (PMC12195895; doi:10.3390/ph18060887)
Supplement: Supplementary file 1 [file pharmaceuticals-18-00887-s001.zip › pharmaceuticals-3652781-supplementary.pdf]

Table S1. The specific characteristic features of each leech species

NO.	leech species	The specific characteristic features
1	*Hirudo nipponica* Whitman	The dorsal surface of HN is light yellow-green to gray-green, with five yellowish-white longitudinal stripes, either continuous or discontinuous, the central stripe being the widest and longest. These yellowish-white stripes divide the dorsal background color into six longitudinal sections. The two central stripes are the broadest, while the two lateral stripes are narrower. The ventral surface is yellowish-white to dark gray-green, with a very fine gray-green longitudinal stripe on each lateral edge, though not very prominent.
2	*Whitmania pigra* Whitman	The dorsal surface of WP ranges from light yellow-green to dark green, with five longitudinal stripes formed by black spots interspersed with light yellow. The central stripe is the darkest and thickest. The ventral surface is brownish-yellow, with wide black-brown longitudinal stripes along each lateral edge, and approximately seven discontinuous black-brown longitudinal stripes between them.
3	*Poecilobdella javanica* Wahlberg	PJ has a broken black-brown longitudinal stripe along the center of the back, consisting of approximately 22 segments. Between each pair of segments, four black-brown spots are faintly arranged in a square pattern.
4	*Poecilobdella manillensis* lesson	The dorsal surface of PM has a wide gray longitudinal stripe in the center, with four smaller, fine stripes composed of dots on either side of it. The dorsal lateral edges each display a row of large, distinct black spots, alternating with one or two body rings. The ventral surface lacks spots and longitudinal stripes, though the lateral edges feature a wide, prominent black-brown band.
5	*Mimobdella Japonica* Blanchard	MJ exhibits no discernible characteristic stripes or patterns on the dorsal, ventral surfaces, or lateral margins.
6	28	28 has scattered black spots on its ventral surface, without forming longitudinal stripes. Its other characteristics are consistent with WP.

Table S2. The detailed information of the 28 leech samples

NO.	Origin	Location	Lifestyle	Processing method	Collection time	Provider
1	HN	Jining County, Jining City, Shandong Province	wild	Sun-dried	2022.10	Qinglong Meng
2	HN	Sucheng District, Suqian City, Jiangsu Province	wild	Sun-dried	2022.09	Lin Zhu
3	HN	Bamen Town, Baodi District, Tianjin City	wild	Sun-dried	2022.11	Huoyuan Yin
4	HN	Xihua County, Zhoukou City, Henan Province	Wild	Sun-dried	2022.10	Yu Zhang
5	HN	Jining County, Jining City, Shandong Province	cultured	Sun-dried	2022.10	Qinglong Meng
6	HN	Sucheng District, Suqian City, Jiangsu Province	cultured	Sun-dried	2022.09	Lin Zhu
7	HN	De'an County, Jiujiang City, Jiangxi Province	cultured	Sun-dried	2022.09	Xiaoguo Yin
8	HN	Yuanjiang City, Yiyang City, Hunan Province	cultured	Sun-dried	2022.07	Xing Ouyang
9	HN	Jining County, Jining City, Shandong Province	cultured	low-temperature dried	---	Sichuan Qian Yuan Traditional Chinese Medicine slices Co., LTD
10	HN	Gong'an County, Jingzhou City, Hubei Province	cultured	Sun-dried	---	Jingzhou Min Kang Biotechnology Co., LTD
11	HN	Gong'an County, Jingzhou City, Hubei Province	cultured	low-temperature dried	---	Jingzhou Min Kang Biotechnology Co., LTD
12	HN	Bamen Town, Baodi District, Tianjin City	Wild	Sun-dried	2022.11	Huoyuan Yin
13	WP	Shandong (purchased from Anguo Medicinal Materials Market)	wild	Sun-dried	---	Hebei Jinhui Pharmaceutical Co., LTD
14	WP	Jining County, Jining City, Shandong	wild	Sun-dried	2022.10	Qinglong Meng

		Province				
15	WP	Sucheng District, Suqian City, Jiangsu Province	wild	Sun-dried	2022.09	Lin Zhu
16	WP	Bamen Town, Baodi District, Tianjin City	wild	Sun-dried	2022.09	Huoyuan Yin
17	WP	Xihua County, Zhoukou City, Henan Province	wild	Sun-dried	2022.09	Yu Zhang
18	WP	Jining County, Jining City, Shandong Province	cultured	Sun-dried	2022.10	Qinglong Meng
19	WP	Sucheng District, Suqian City, Jiangsu Province	cultured	Sun-dried	2022.09	Lin Zhu
20	WP	De'an County, Jiujiang City, Jiangxi Province	cultured	Sun-dried	2022.08	Xiaoguo Yin
21	WP	Yuanjiang City, Yiyang City, Hunan Province	cultured	Sun-dried	2022.07	Xing Ouyang
22	WP	Gong'an County, Jingzhou City, Hubei Province	cultured	Sun-dried	---	Jingzhou Min Kang Biotechnology Co., LTD
23	WP	Gong'an County, Jingzhou City, Hubei Province	cultured	low-temperature dried	---	Jingzhou Min Kang Biotechnology Co., LTD
24	PJ	Qinnan District, Qinzhou City, Guangxi Province	wild	Sun-dried	2022.09	Chunjin Zhu
25	PM	Zhongshan City, GuangDong Province	cultured	low-temperature dried	2023.09	Sichuan Qian Yuan Traditional Chinese Medicine slices Co., LTD
26	MJ	Xihua County, Zhoukou City, Henan Province	wild	Sun-dried	2022.10	Yu Zhang
27	MJ	Xinhui District, Jiangmen City, Guangdong Province	wild	Sun-dried	2022.08	Fencheng Zhang
28	Unidentified counterfeit	De'an County, Jiujiang City, Jiangxi Province	cultured	Sun-dried	2022.09	Xiaoguo Yin

---: uncertain time

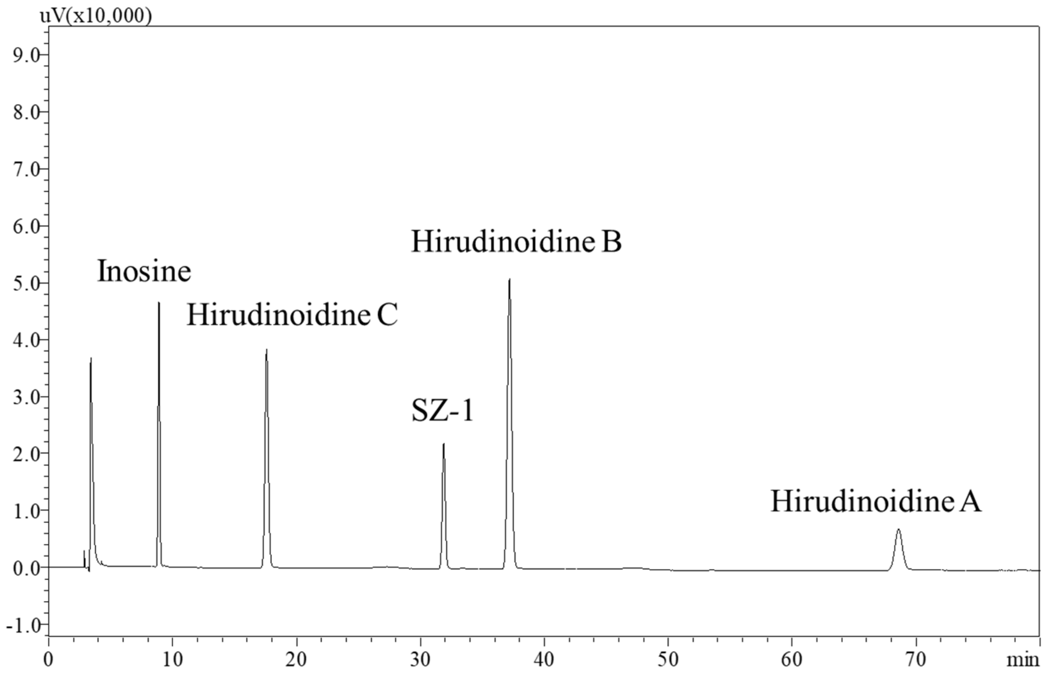

Figure S1. HPLC profile of a mixture of reference substance SZ–1, hirudamine A, hirudamine B, hirudamine C, and inosine

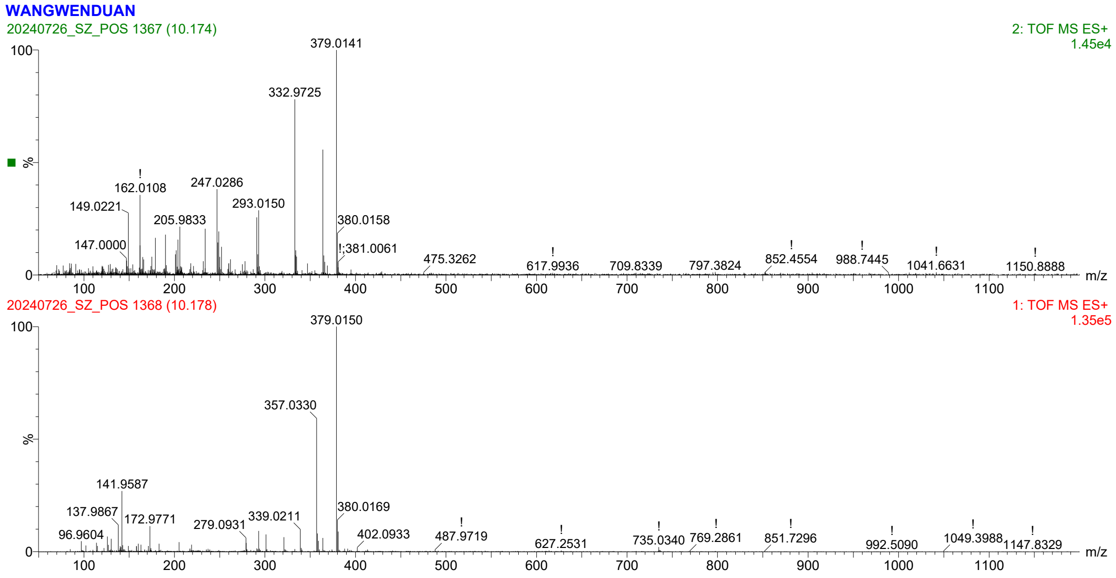

Figure S2. MS spectrum of the fragments and precursor of SZ–1

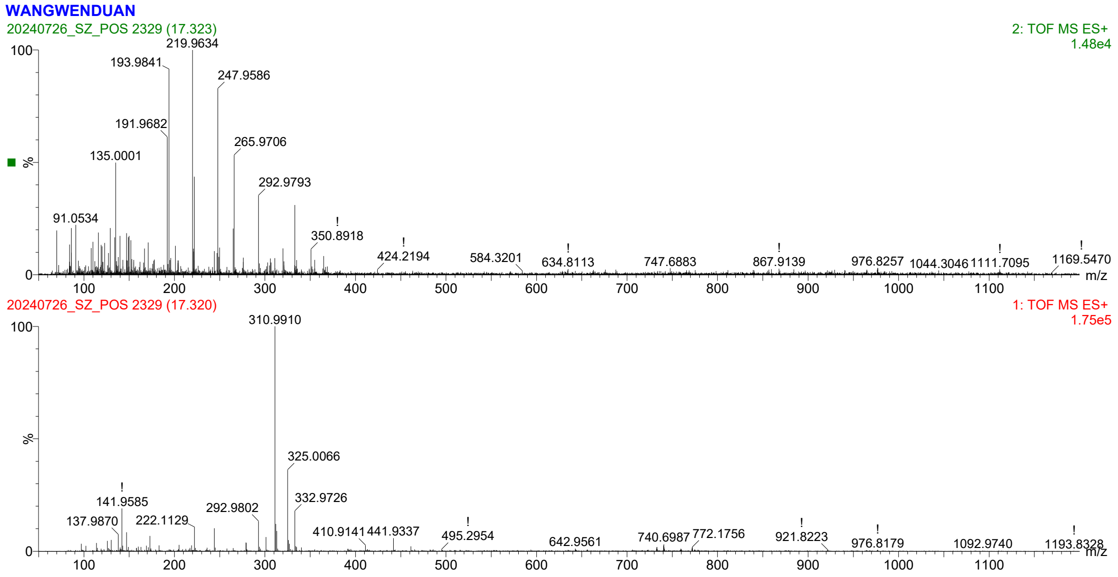

Figure S3. MS spectrum of the fragments and precursor of hirudamine A

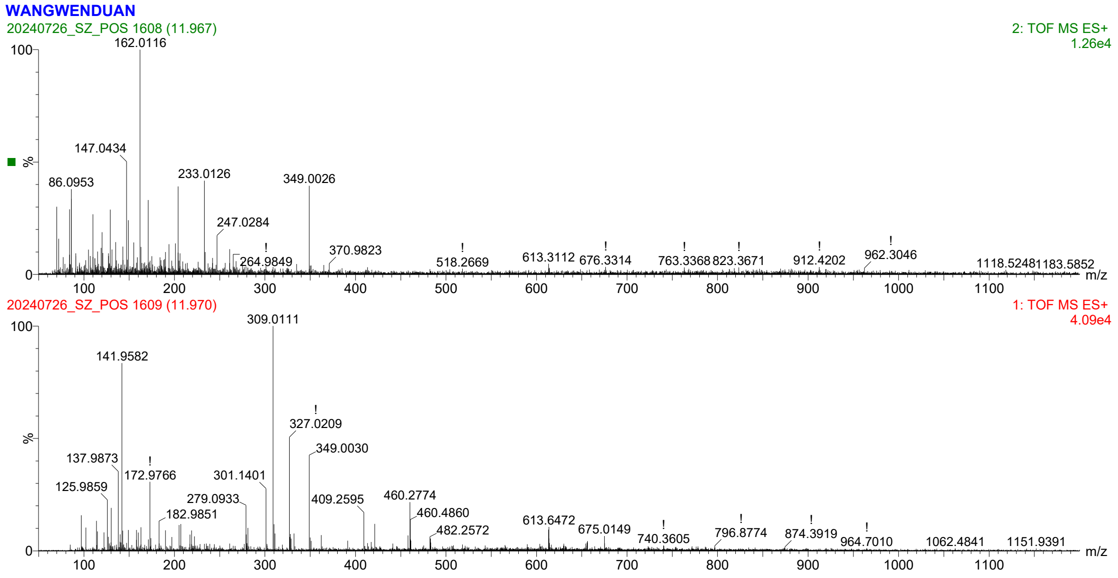

Figure S4. MS spectrum of the fragments and precursor of hirudamine B

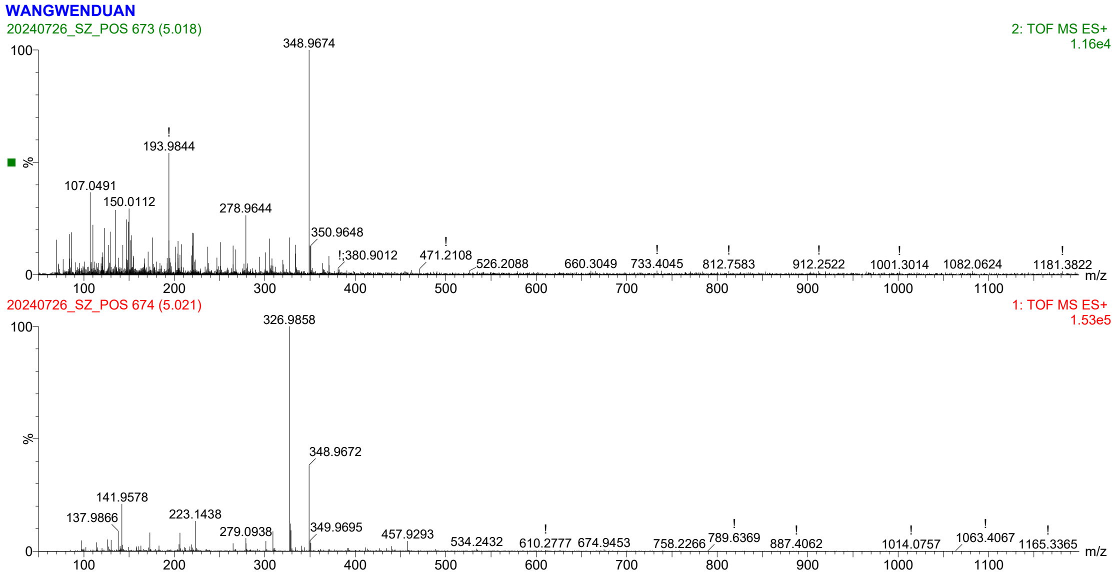

Figure S5. MS spectrum of the fragments and precursor of hirudamine C

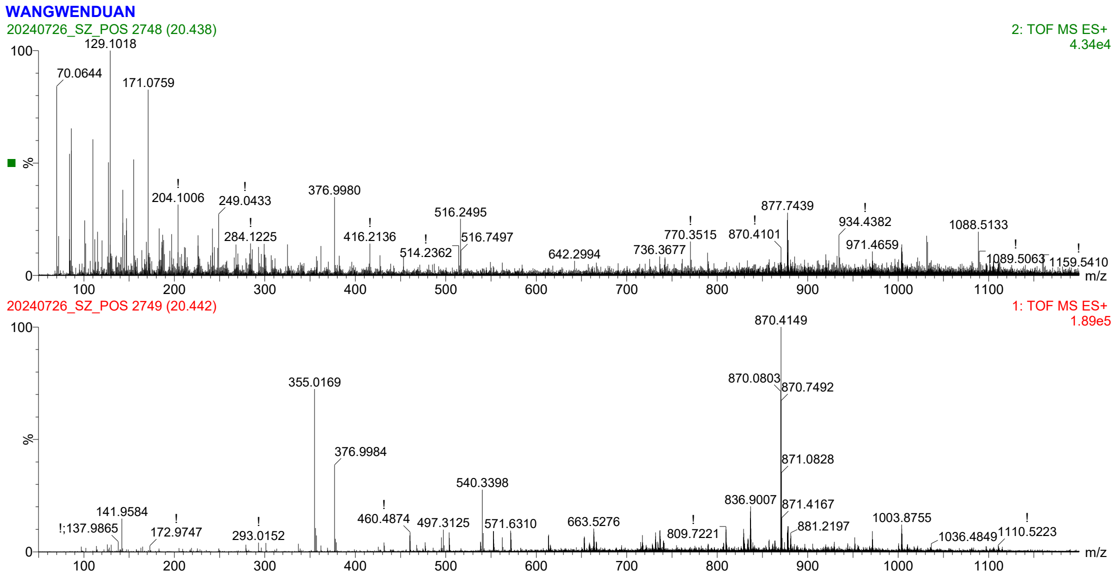

Figure S6. MS spectrum of the fragments and precursor of whitmanines B

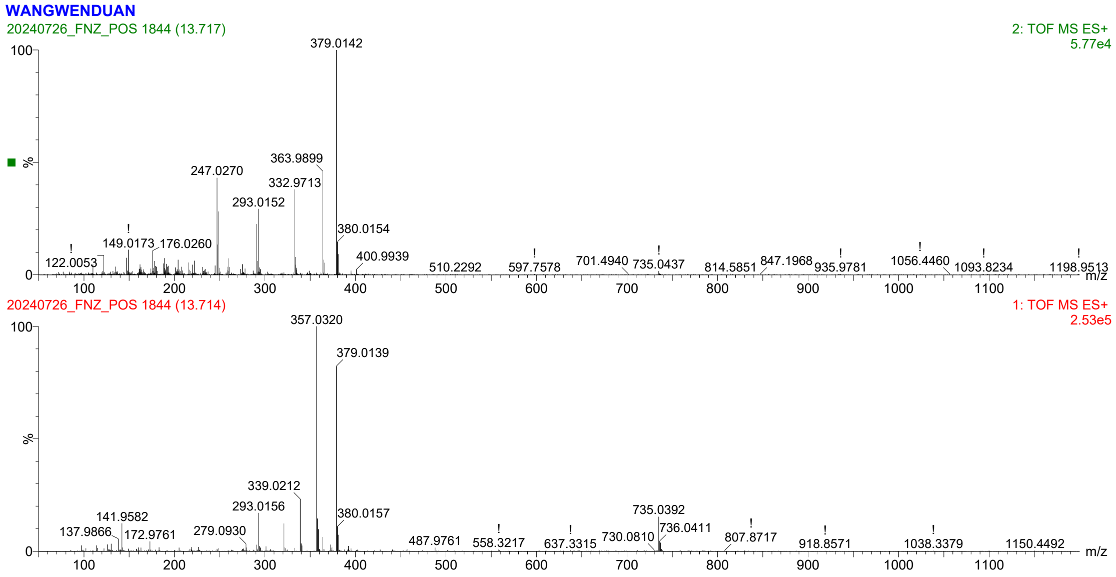

Figure S7. MS spectrum of the fragments and precursor of poecilobdellasulfide B

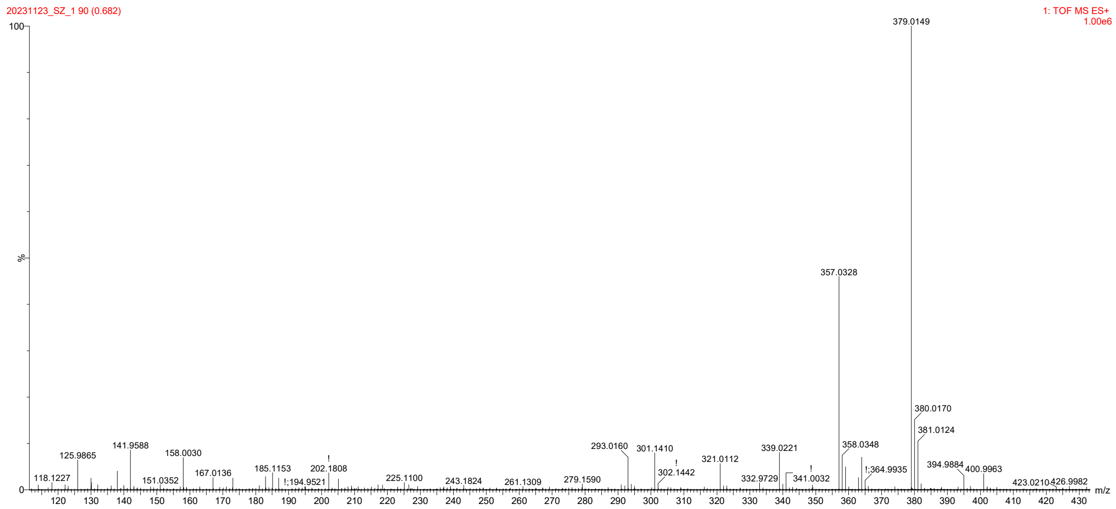

Figure S8. *HR-ESI-MS spectrum of* SZ–1

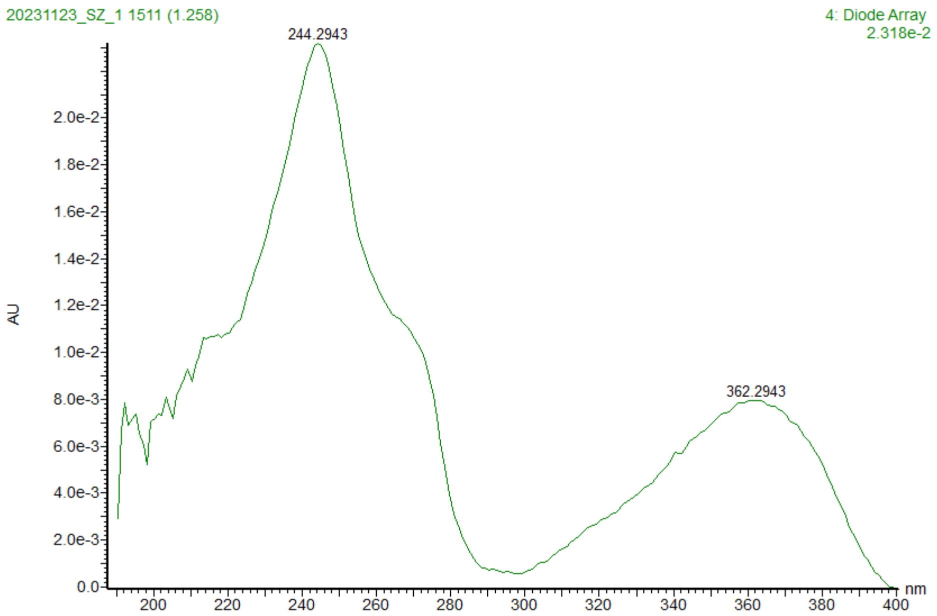

Figure S9. UV spectrum of SZ–1

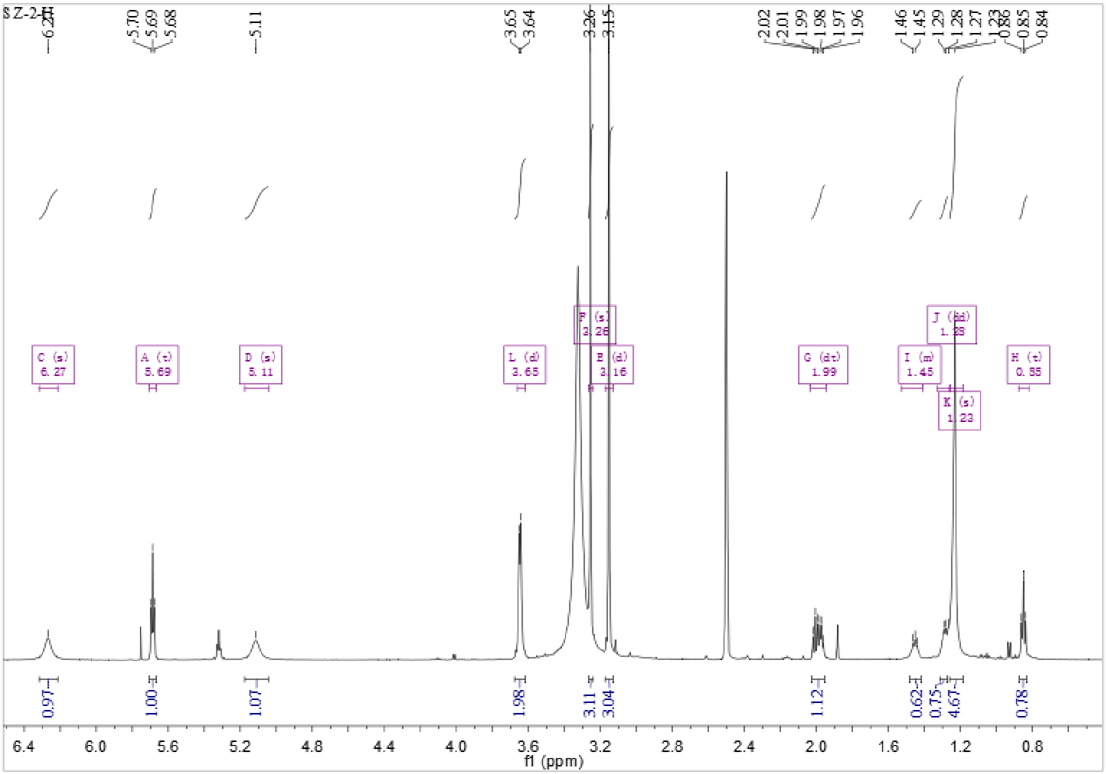

Figure S10. *^{1}H NMR spectrum of* SZ–1 in *DMSO-d_6*

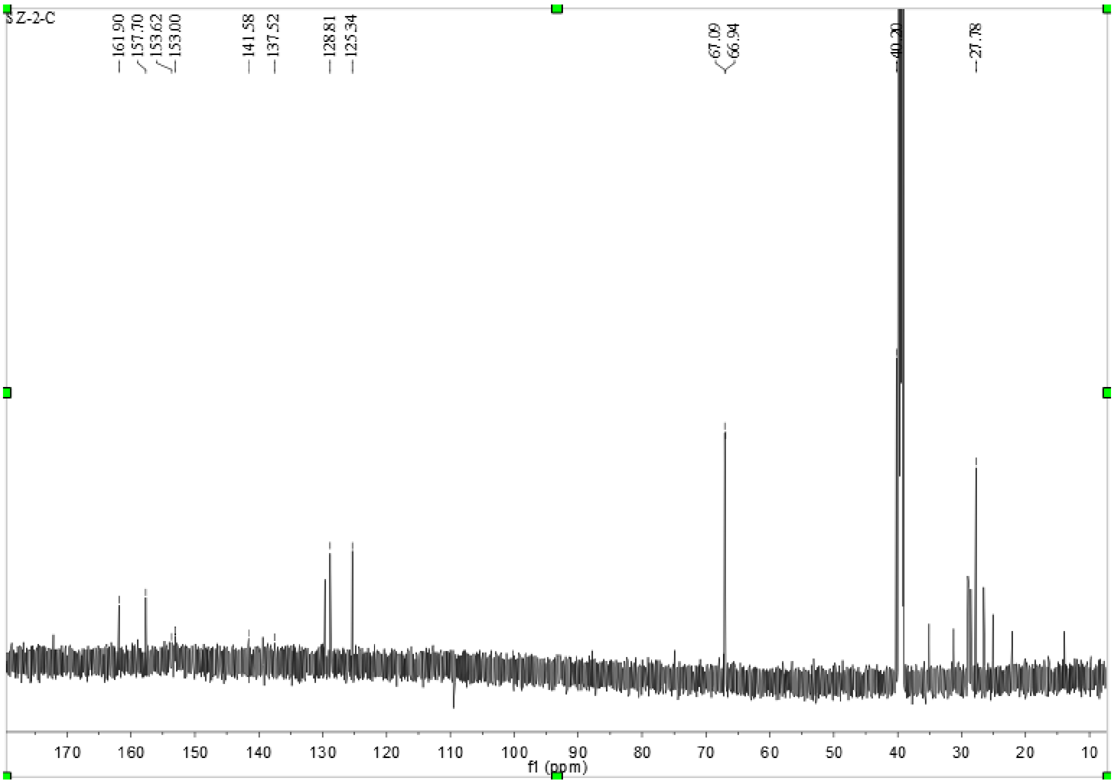

Figure S11. *^{13}C NMR spectrum of* SZ–1 in *DMSO-d_6*

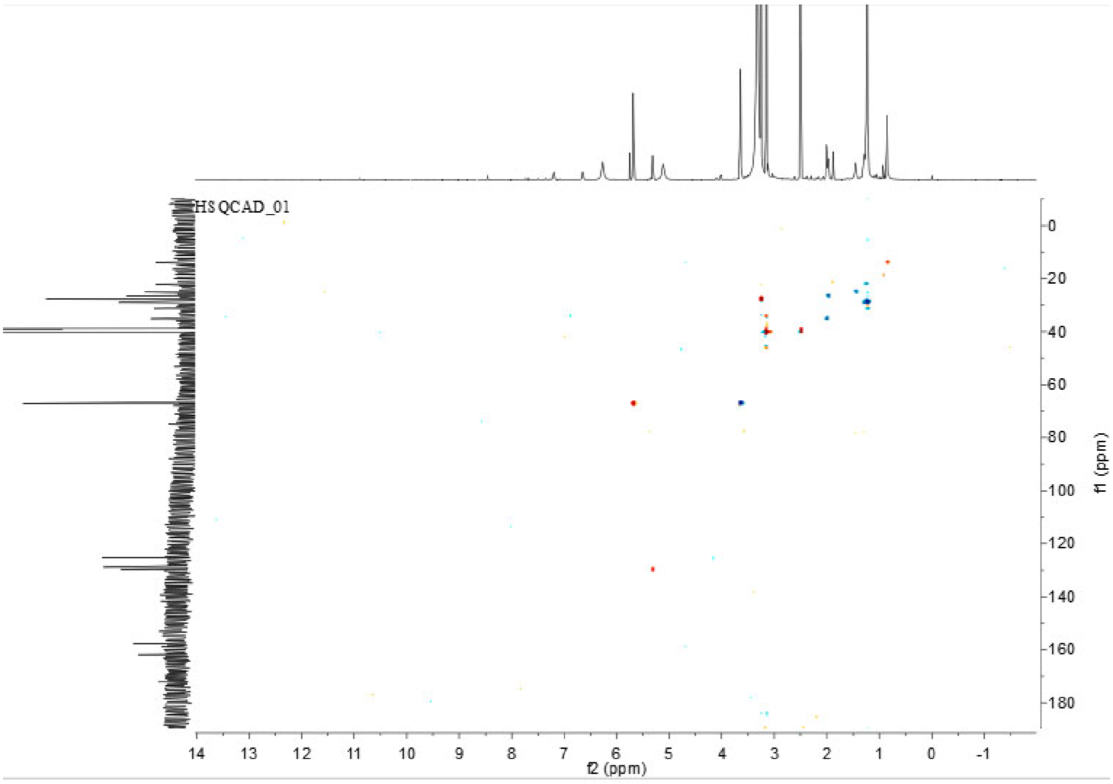

Figure S12. *HSQC spectrum of* SZ–1 in *DMSO-d_6*

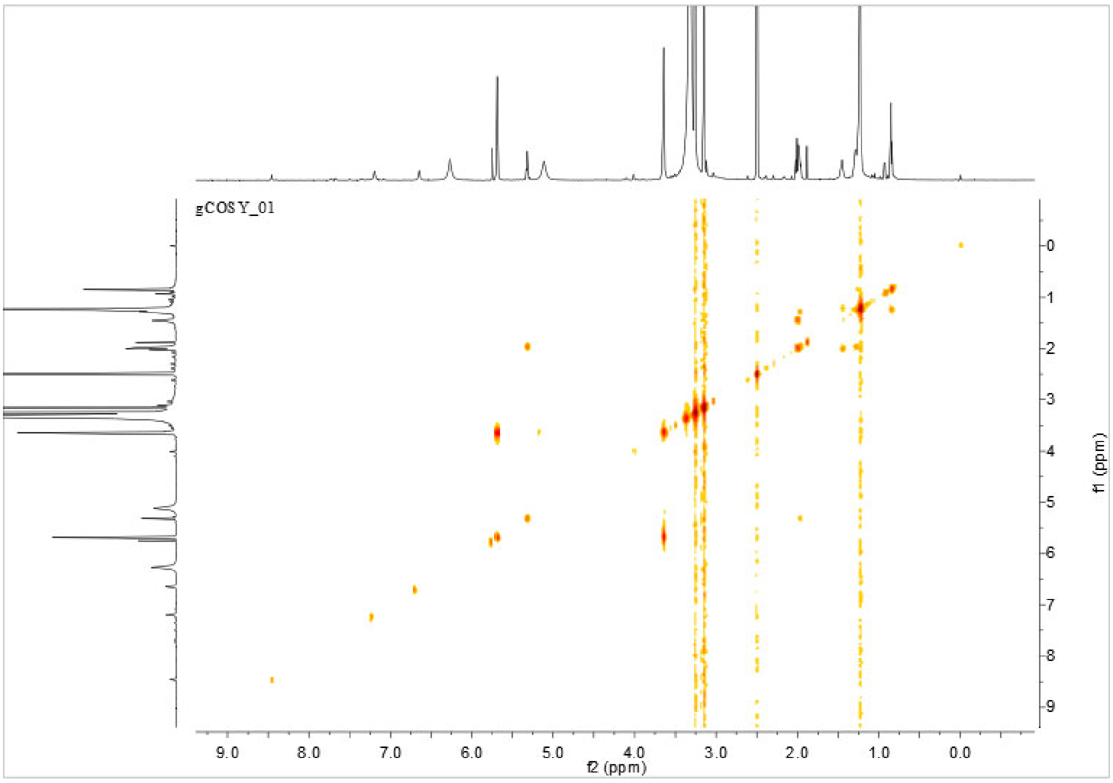

Figure S13. *^{1}H-^{1}H COSY spectrum of* SZ–1 in *DMSO-d_6*

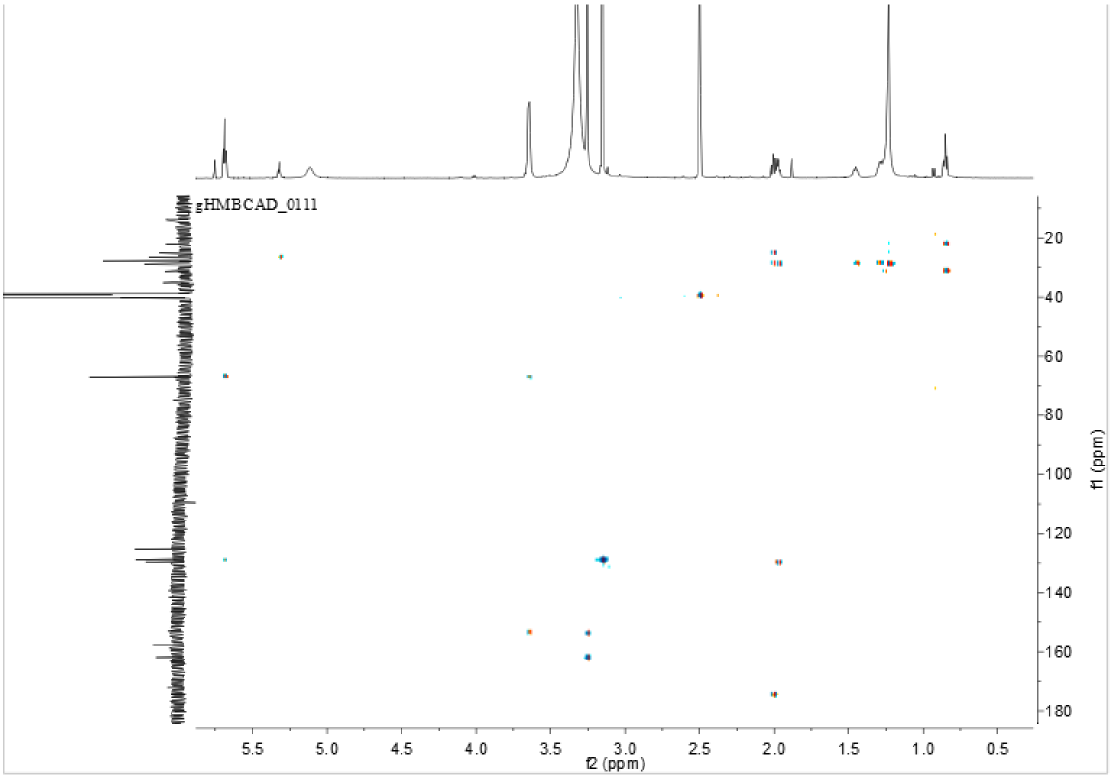

Figure S14. *HMBC spectrum of* SZ–1 in *DMSO-d_6*

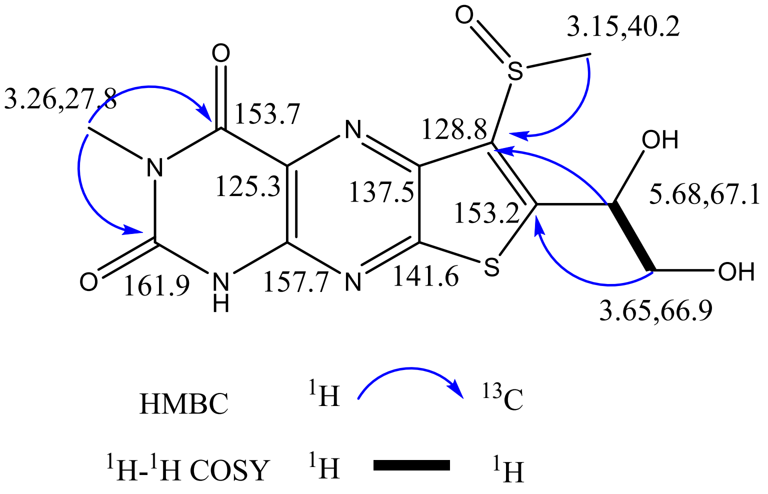

Figure S15. Key *^{1}H-^{1}H COSY* and *HMBC* correlations of SZ–1

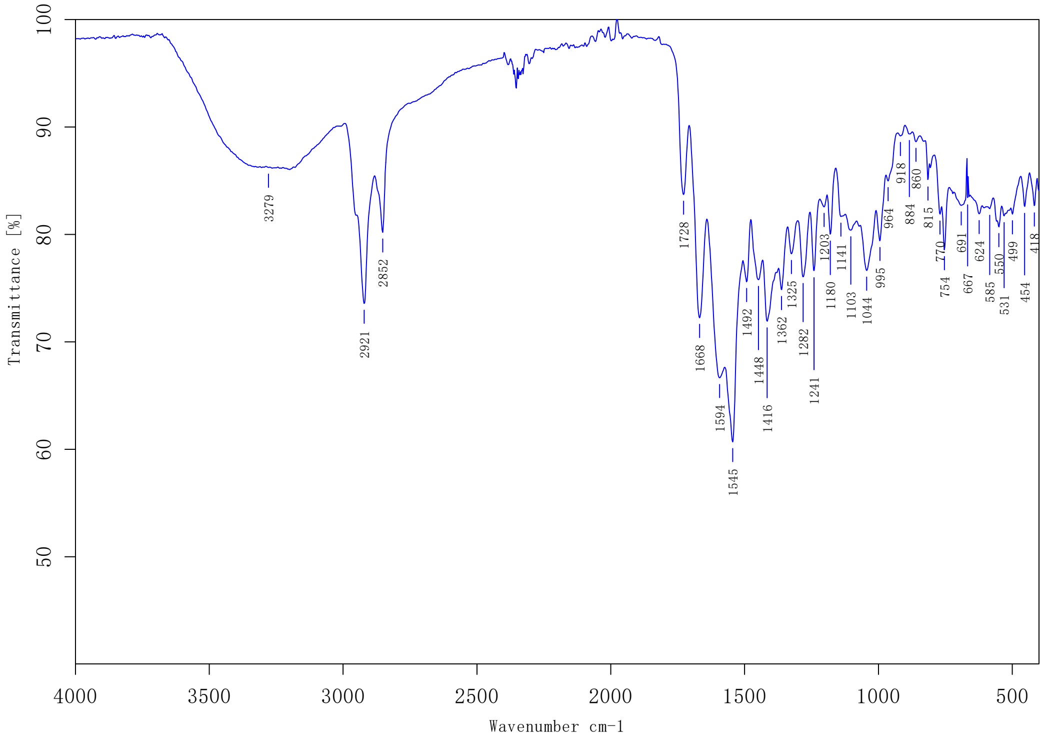

Figure S16. *IR spectrum of* SZ–1

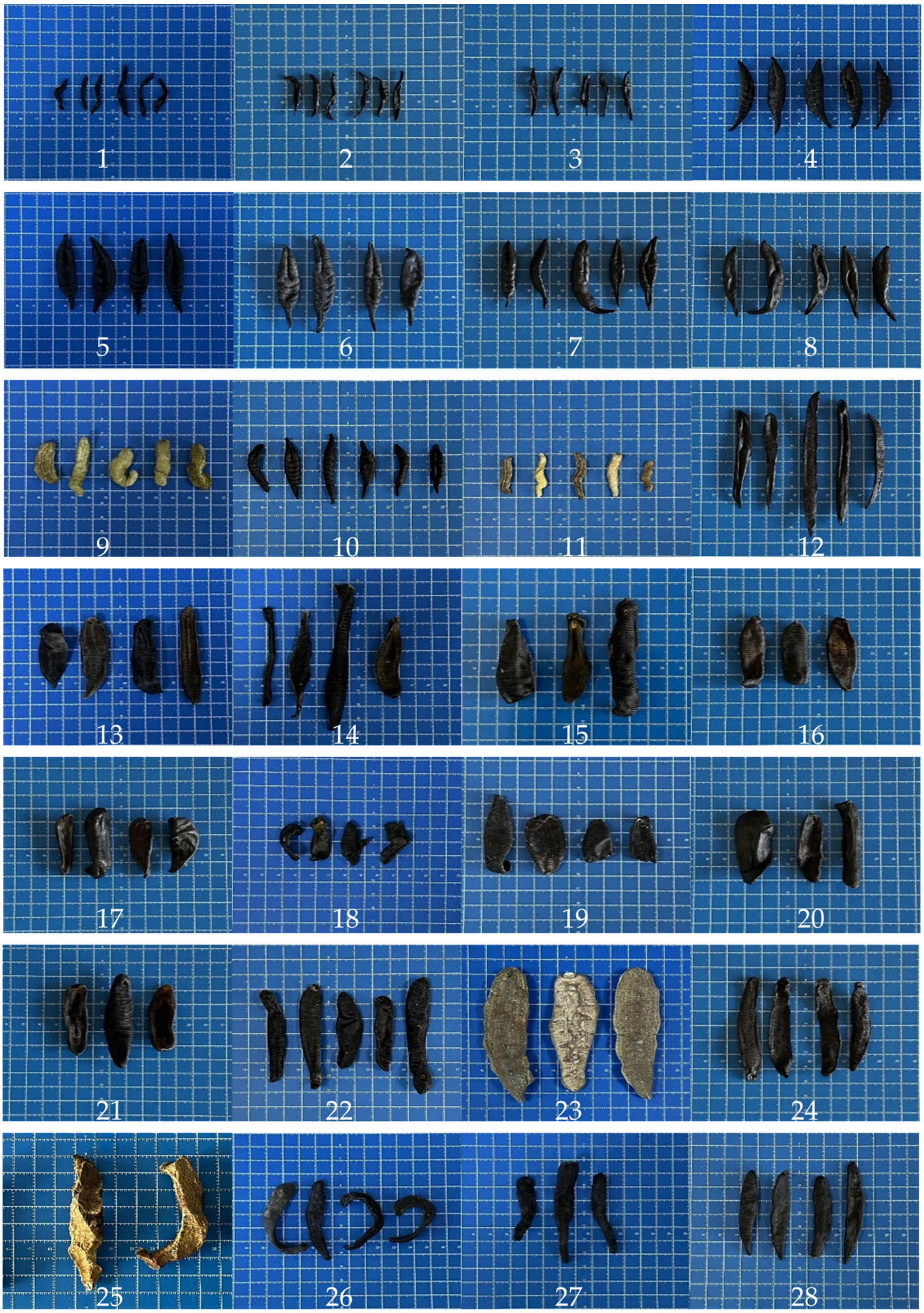

Figure S17. The leeches' pictures

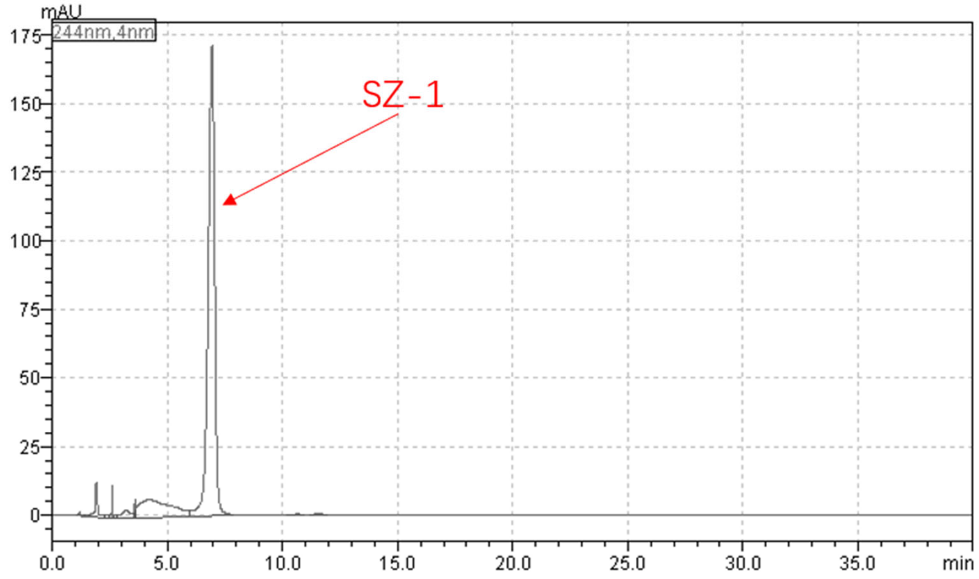

Figure S18. HPLC spectrum of SZ–1
